# Enzyme‐mediated remodeling of the extracellular matrix and glycocalyx to enhance immunotherapy in solid tumors

**DOI:** 10.1002/btm2.70180

**Published:** 2026-09-28

**Authors:** Navid Mirmohammadsadegh, Mohsen Amin

**Affiliations:** ^1^ Department of Drug and Food Control, Faculty of Pharmacy Tehran University of Medical Sciences Tehran Iran; ^2^ Nutraceutics Research Center Tehran University of Medical Sciences Tehran Iran

**Keywords:** ECM‐targeting enzymes, extracellular matrix (ECM), glycocalyx, immunotherapy, microbial‐derived enzymes, solid tumors, tumor microenvironment (TME)

## Abstract

The tumor‐associated extracellular matrix (ECM) and glycocalyx form a dynamic barrier that provides physical shielding and immunosuppressive signaling, leading to therapeutic resistance. A growing body of evidence supports enzyme‐based strategies to remodel these barriers and improve therapeutic access to tumors. This review examines enzymes that act as “path cleansers” by cleaving components of the ECM or the glycocalyx to restore the efficacy of immunotherapies. Moreover, some enzymes may exert effects beyond barrier remodeling, including modulation of signaling pathways, enhancement of immune responses, or even direct effects on the tumor. In this review, barrier‐remodeling enzymes, categorized into different classes, are discussed as potential adjuncts to cancer immunotherapy. This review also highlights the bioengineering approaches and the delivery strategies used to enhance tumor specificity, efficacy, and safety. While such therapeutic strategies show promising outcomes, the evidence hierarchy and potential translational risks are also discussed. Improved patient stratification and computationally guided enzyme discovery and protein engineering, including strategies to reduce immunogenicity, can facilitate the clinical translation of enzyme‐assisted immunotherapy.

**Translational Impact Statement**

Dense extracellular matrix and glycocalyx barriers in solid tumors limit therapeutic and immune‐cell access to tumors and promote immunosuppressive signaling, undermining immunotherapy efficacy. Enzyme‐based remodeling strategies act as “path cleansers” to degrade these barriers, enhance therapeutic penetration, and modulate antitumor immunity. This review outlines key enzymes, bioengineering innovations, and targeted delivery approaches and evaluates translational challenges, aiming to advance enzyme‐enabled combinatorial immunotherapies toward clinical application in resistant solid tumors.

## 1 INTRODUCTION

The cellular and molecular building blocks of a tumor milieu include tumor cells, immune cells, fibroblasts, and other tissue‐specific cells, all of which are held together by the extracellular matrix (ECM). The ECM framework provides physical and biochemical support for cancer cells. In general, ECM consists of key components, including collagens, proteoglycans, glycosaminoglycans, elastin, laminins, fibronectin, and various other glycoproteins.^1^ In healthy tissues, ECM composition and stiffness are finely tuned to maintain homeostasis. In contrast, solid tumors exploit the ECM to their advantage to different extents. The predominant view of the cancerous ECM as a mere static “wall” has gradually shifted to the paradigm that it acts as a dynamic scaffold that not only affects cells' behavior within the tumor microenvironment (TME) but also regulates several signaling pathways.^2^ Tumors remodel the ECM to facilitate immune evasion, promote metastasis, and cause therapeutic resistance.^2^ Apart from obstructing therapeutic trafficking, ECM remodeling and densification lead to hypoxia, which consequently contributes to resistance to chemotherapy and radiotherapy.^3^ Moreover, hypoxic conditions in dense ECM can lead to an upregulation of immunosuppressive factors within TME that alter the response to immunotherapies.^4^ Despite these cumulative malignant features, immunotherapies remain among the most promising strategies for cancer treatment. However, progressive ECM remodeling remains a central barrier to immunotherapy efficacy in solid tumors, underscoring the need for strategies that directly address ECM‐mediated resistance.^5^ Additionally, abnormal accumulation of ECM components dampens the local immune responses elicited by therapeutics.^5^, ^6^ Several suppressive signaling pathways, initially triggered by the ECM and/or ECM‐associated components, are harnessed by cancer cells. This phenomenon consequently leads to the curbed recruitment of immune cells into the TME.^7^ Furthermore, fortified barriers in solid tumors extend beyond the ECM; the tumor‐associated glycocalyx, often enriched in mucins and hypersialylated glycans, promotes immune evasion through physical shielding and immunoregulatory signaling^8^ (Figure 1a). By recognizing the footprints of tumor ECM/glycocalyx composition and stiffness in cancer progression and therapeutic effectiveness, researchers have begun to reevaluate their roles in tumors and to use them as potential targets for drug development.^9^ One emerging approach involves using enzymes that can act as “path cleansers,” cutting through components to pave the way for therapeutics and alter signaling pathways (Figure 1b). However, ECM/glycocalyx remodeling should not be viewed simply as dismantling a passive physical barrier. Realistically, these extracellular structures regulate immune trafficking, stromal signaling, vascular integrity, and metastatic niche formation. In this regard, using path cleansers may act as a double‐edged sword that should cut the extracellular barrier in a controlled fashion to avoid excessive or off‐target remodeling. The potential risks have been summarized in Box 1.

**FIGURE 1:**
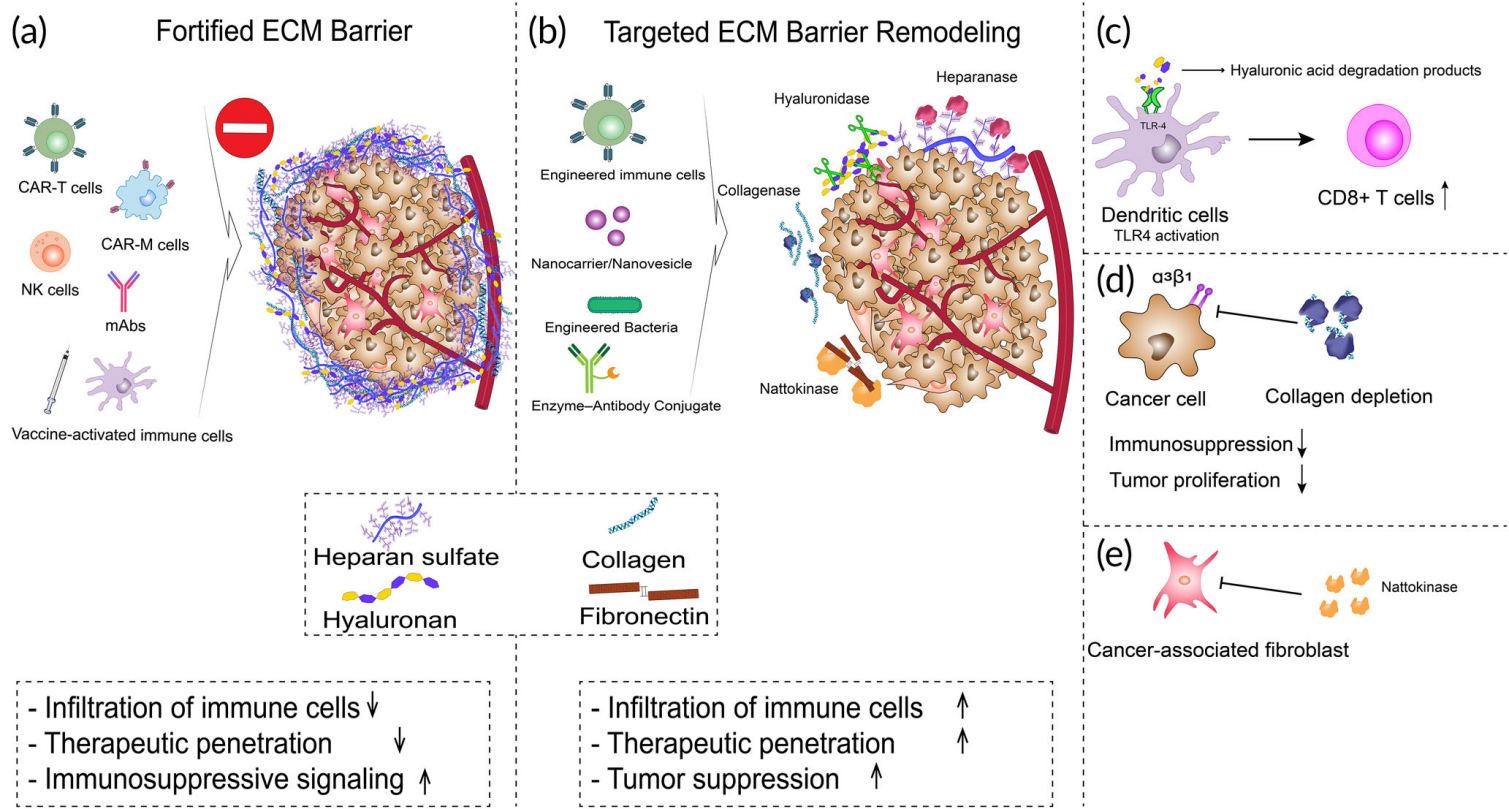
Targeted enzymatic extracellular matrix (ECM) editing improves immune access and potentiates immunotherapy through both physical de‐obstruction and immunomodulatory effects. (a) Illustrate how a fortified ECM barrier physically restricts immune cell infiltration and therapeutic penetration while promoting immunosuppressive signaling; (b) targeted ECM barrier editing using ECM‐editing enzymes improves immune access and drug distribution. Enzymes can be delivered via engineered immune cells, nanocarriers, engineered bacteria, or enzyme‐antibody conjugates to potentiate immunotherapies (e.g., chimeric antigen receptor [CAR]‐T cells, CAR macrophages, natural killer (NK) cells, monoclonal antibodies, and cancer vaccines). The activity of enzymes may go beyond physical barrier removal, including (c) immune activation by hyaluronan degradation products, (d) reduced tumor‐promoting oncogenic collagen‐integrin signaling following collagen depletion, and (e) indirect inhibition of cancer‐associated fibroblasts upon activation of nattokinase.

BOX 1 **Potential consequences of extracellular enzymatic remodeling**

The “path cleansers” constitute a variety of biological activities discussed above. However, there are possible adverse reactions that should be taken into consideration as potential drawbacks of the whole strategy. Some examples of such issues are summarized as follows:

- *Metastasis promotion*: Excessive extracellular matrix (ECM) degradation may facilitate tumor‐cell invasion or dissemination in some settings. This concern may be particularly relevant to heparanase (HPSE) and collagenases.^10^, ^11^
- *Vascular remodeling*: Degradation of vascular or perivascular matrix may alter microvascular function or barrier properties, with collagenases and HPSE warranting particular consideration.^11^, ^12^
- *Tissue damage*: Off‐target degradation of ECM or glycocalyx components may damage normal tissues. This concern is potentially relevant across the enzyme classes discussed here, particularly when their substrates are widely distributed, or enzyme activity is insufficiently localized.^13^, ^14^
- *Immune dysregulation*: ECM remodeling may generate bioactive fragments or release matrix‐bound mediators, producing context‐dependent immune effects. HPSE and hyaluronidases are relevant examples, while excessive sialidase activity may perturb glycan‐dependent immune interactions.^15^, ^16^

Direct comparative safety data remain limited; thus, these represent mechanistic concerns rather than an established risk ranking.

In this narrative review, we focus on enzymes that can remodel tumor ECM/glycocalyx to enhance the efficacy of immunotherapies. We categorized these strategies by enzyme classes, highlighting how selective degradation of specific extracellular components could alleviate tumor barriers that would otherwise hinder immune cell infiltration and drug delivery. Furthermore, this review covers recent advances in enzyme delivery and bioengineering approaches, with attention to enzyme‐specific limitations and translational considerations. In addition to the well‐studied enzymes, we highlight potential efforts in the discovery and “repurposing” of novel enzymes from different sources, including eukaryotic and prokaryotic organisms, to expand the barrier‐editing toolkit for immunotherapy. The term “repurposing” has been coined for medications with specific therapeutic use that are useful for other indications; we are using this term for such enzymes in this article. The available evidence is heterogeneous and spans in vitro studies, animal models, and limited clinical investigations, with most strategies remaining at the preclinical stage.

## 2 HEPARANASE

Heparan sulfate is a sulfated glycosaminoglycan (GAG) and a constituent of the ECM, where it contributes to the regulation of cell trafficking.^17^ Many immune effector cells require access to their site of action to mount an effective response. This is orchestrated through a combination of adhesion dynamics, cytoskeletal remodeling, and localized enzymatic degradation of ECM components, which together facilitate immune cell migration.^18^ Among such enzymes, heparanase (HPSE) is the only mammalian enzyme capable of cutting the heparan sulfate chains of heparan sulfate proteoglycans (HSPGs) within the ECM.^19^ HSPGs regulate cell signaling, tissue architecture, and immune responses. Such functions become dysregulated in cancer and contribute to ECM stiffening, impeding the infiltration of immune cells.^20^ Upon activation, immune cells, including T cells, neutrophils, monocytes, and natural killer (NK) cells, can produce HPSE.^21^, ^22^ However, under certain conditions, immune cells may exhibit reduced or lost HPSE expression, limiting their ability to penetrate the dense ECM of solid tumors.^23^, ^24^

HPSE has also been widely studied as a target for inhibition due to its pro‐tumorigenic functions, including the promotion of metastasis and angiogenesis.^25^ However, when restricted to therapeutic immune cells or localized to the tumor interface, HPSE‐mediated ECM remodeling may instead enhance immune‐cell infiltration and improve immunotherapy efficacy.^23^, ^24^

In recent years, chimeric antigen receptor (CAR)‐T cell therapy has garnered increasing attention as a promising treatment for cancer. However, these engineered cells have shown limited effectiveness against solid tumors due to the immunosuppressive and physically restrictive TME, as well as challenges associated with their manufacturing.^26^ The general workflow for CAR‐T cell manufacturing includes ex vivo expansion of the cells. During this process, especially if prolonged, the cells may undergo phenotypic alterations that can impact therapeutic outcomes.^27^ For example, long‐term ex vivo‐expanded T cells (LTE‐T cells) exhibit downregulated HPSE expression, potentially due to p53‐mediated repression; however, freshly isolated T cells retain their ability to express HPSE.^23^ These observations have prompted the exploration of HPSE reconstitution as a strategy to restore ECM‐degrading capacity and migratory competence. A preclinical study investigated CAR‐T cells co‐expressing tumor‐specific CARs and HPSE to assess their performance in ECM‐rich solid tumor models. CAR^+^ LTE‐T cells, with or without HPSE expression, showed a comparable cytolytic profile against antigen‐positive tumor cells in the absence of ECM. On the other hand, CAR^+^ LTE‐T cells armed with HPSE revealed robust tumor‐eliminating activity in the presence of ECM. In vivo, HPSE‐expressing CAR‐T cells displayed enhanced intratumoral accumulation, superior tumor control, and prolonged survival compared with HPSE‐deficient counterparts, without evidence of abnormal accumulation in the evaluated non‐target organs (lung and liver).^23^

Importantly, the reduction of HPSE expression is not limited to T cells. Ex vivo studies have shown that the expansion of NK cells can also lead to decreased HPSE expression. Consistent with this observation, HPSE‐deficient NK cells exhibit markedly impaired infiltration into tumor spheroids compared with HPSE‐competent counterparts, reinforcing the role of HPSE as a key mediator of immune cell penetration through the ECM. To restore diminished ECM‐degrading capacity, a bioengineering approach was implemented to increase HPSE activity in NK cells by expressing a membrane‐anchored, enzymatically active HPSE construct.^24^ Because elevated HPSE expression in certain tumors is associated with increased invasion and metastatic potential, anchoring HPSE to the NK cell surface as an integral membrane protein may help minimize unintended effects that could otherwise promote tumor invasion and angiogenesis.^10^, ^24^ However, whether this design limits systemic dissemination of enzymatic activity was not directly evaluated. Interestingly, such engineered NK cells did not exhibit compromised activity despite the introduction of synthetic HPSE constructs. Moreover, HPSE‐expressing NK cells demonstrated enhanced penetration into tumor spheroids, whereas control cells largely remained confined to peripheral regions, indicative of limited invasive capacity. An in vivo tumor model further supported these findings, demonstrating that HPSE‐engineered NK cells achieve better tumor infiltration and enhanced tumor control compared with unmodified NK cells.^24^

The potential effects of HPSE beyond immune cells also warrant consideration, although the consequences of therapeutic HPSE‐mediated remodeling for nonimmune stromal compartments remain poorly characterized. In endothelial cells, addition of purified HPSE increased migration and invasion. Notably, HPSE can exert biological effects through both HS‐degrading enzymatic and non‐enzymatic mechanisms.^28^ In tumor models, changes in HPSE expression also altered heparan sulfate distribution around blood vessels and pericyte coverage, suggesting that HPSE‐dependent remodeling may influence the perivascular compartment. In addition to its direct effects, HPSE activity on heparan sulfate can release bound growth factors, cytokines, and chemokines, potentially affecting neighboring stromal cells.^19^ However, whether localized HPSE expression by engineered immune cells produces comparable stromal effects remains unknown.

Despite encouraging enzymatic activity, HPSE engineering lacks support beyond preclinical evidence, and its pleiotropic effects further emphasize the need for spatial and temporal control of HPSE activity. For example, engineered CAR‐T and NK cell studies reported improved infiltration without overt short‐term pathology in selected models. Yet, persistent systemic activity, off‐tumor heparan sulfate degradation, vascular remodeling, or metastasis‐promoting effects have not been comprehensively evaluated.^23^, ^24^, ^29^ Therefore, the absence of reported adverse effects should not be interpreted as evidence of safety. Additional safety strategies should also aim to restrict HPSE activity to the target tissue and limit prolonged or systemic exposure. Membrane anchoring, as used in the NK cell platform described above, represents one such strategy^24^; however, emerging synthetic‐biology tools may offer further opportunities to regulate HPSE through inducible circuits, antigen‐responsive expression systems, logic gates, and safety switches.^30^ These regulatory approaches have been explored in engineered cells to regulate CAR expression, cytokine production, or other cellular functions.^30^ Translating these approaches to HPSE could help restrict its expression to defined conditions and reduce the risks associated with constitutive or off‐target activity.

## 3 HYALURONIDASE

Hyaluronan (HA) is a linear, non‐sulfated GAG that, in addition to its structural roles within the ECM, serves as a ligand for several receptors, particularly those involved in immune responses and cancer progression.^31^, ^32^ HA‐rich tumors form a physical barrier that impairs the therapeutic outcomes of biopharmaceuticals by limiting their penetration. Notably, studies have shown that HA accumulation decreases access to chemotherapeutic agents as well.^33^, ^34^, ^35^ High HA accumulation in HER2^+^ breast tumors and epidermal growth factor receptor (EGFR^+^) head and neck squamous cell carcinomas may contribute to resistance to trastuzumab and cetuximab, respectively, by limiting antibody access. In addition to limiting antibody penetration, the HA‐rich TME hampers NK cell migration toward tumor cells, disrupting the formation of synapses essential for antibody‐dependent cell‐mediated cytotoxicity (ADCC), ultimately compromising therapeutic efficacy. Moreover, the HA‐fortified matrix can also hinder NK cell‐mediated cytotoxicity (non‐antibody‐mediated cytotoxicity).^36^ Given these challenges posed by HA accumulation, enzymatic depletion of HA has emerged as a rational strategy to remodel the tumor stroma and restore therapeutic accessibility. PH20, a hyaluronidase naturally expressed in human sperm, is highly active and has previously been shown to enhance drug penetration by degrading HA in tumors.^37^ Because unmodified PH20 has a short circulating half‐life, polyethylene glycol (PEG) was used to extend its half‐life, leading to the development of PEGPH20.^33^ Reduced HA content in tumors treated with PEGPH20 led to enhanced antibody‐dependent and NK cell‐mediated cytotoxicity, resulting from improved access. These findings underscore the therapeutic potential of HA‐targeting strategies to overcome stromal barriers and potentiate antibody‐based immunotherapies.^36^

Despite the therapeutic potential of hyaluronidase, concerns similar to those about HPSE still pose significant translational challenges. Whereas HPSE‐based approaches remain largely preclinical, PEGPH20 has advanced to clinical evaluation, providing important lessons regarding the safety and efficacy of systemic PEGPH20 administration. In SWOG S1313, PEGPH20 combined with modified FOLFIRINOX increased gastrointestinal and thromboembolic toxicity, reduced chemotherapy exposure, and was associated with inferior survival.^38^ In HALO 109‐301, adding PEGPH20 to nab‐paclitaxel plus gemcitabine increased the objective response rate but did not improve overall or progression‐free survival despite HA‐high patient selection.^39^ Although elevated endogenous hyaluronidase expression has been associated with tumor progression and metastasis, preclinical studies indicate that the effects of exogenous hyaluronidase administration are context‐dependent and cannot be assumed to promote or inhibit metastasis.^40^, ^41^, ^42^ More recent hyaluronidase strategies, including PH20‐expressing CAR‐T cells, EV‐based delivery, and stimulus‐responsive nanocarriers, have been developed to address the limitations of systemic PEGPH20 administration by restricting enzymatic activity to the tumor, improving local retention, and reducing systemic exposure, off‐target HA degradation, and treatment‐related toxicity.

One such approach involves integrating hyaluronidase into engineered immune cell platforms to facilitate penetration into HA‐rich TMEs. In this context, anti‐mesothelin CAR‐T cells engineered to secrete Fc‐fused PH20 demonstrated enhanced access to tumor cells within HA‐dense matrices without compromising cytotoxicity or cytokine secretion. In a xenograft model using human gastric cancer cells, PH20‐expressing CAR‐T cells exhibited greater tumor regression and T cell infiltration compared to conventional CAR‐T therapy, accompanied by reduced intratumoral HA levels.^43^

To further utilize PH20 in priming tumors for CAR‐T cell activity, anti‐GPC3 CAR‐T cells were engineered to co‐express IL‐7 and PH20, with each component addressing distinct limitations of CAR‐T therapy in solid tumors. IL‐7 expression promoted T cell proliferation, survival, and maintenance of a memory‐like phenotype, while PH20 expression facilitated HA degradation and stromal remodeling, thereby improving CAR‐T cell infiltration into HA‐rich tumor regions. In hepatocellular carcinoma in vitro models, dual‐engineered CAR‐T cells exhibited reduced apoptosis and enhanced proliferative capacity during prolonged tumor exposure. In vivo, these properties translated into improved intratumoral accumulation, greater tumor regression, prolonged survival, and reduced tumor relapse in rechallenge models.^44^

In addition to genetic engineering, bioorthogonal chemistry has enabled rapid and modular functionalization of CAR‐T cells with hyaluronidase, thereby obviating the need for extensive ex vivo manipulation, which can cause undesirable alterations in cells. Researchers used bioorthogonal click chemistry to conjugate hyaluronidase to CAR‐T cells (CD19‐targeted and carcinoembryonic antigen [CEA]‐targeted) to confer ECM‐degrading properties. Moreover, to counteract immunosuppressive signaling within the TME, anti‐programmed death‐ligand 1 (PD‐L1) was additionally conjugated via acidity‐responsive linkers, enabling selective release under acidic TME conditions. Dual modification (H‐P@CAR‐T) preserved CAR‐T key markers and cytotoxic function, indicating minimal impact on cellular integrity. Notably, hyaluronidase‐modified CD19‐targeted CAR‐T cells demonstrated enhanced infiltration through ECM‐mimicking gels and ex vivo A20 tumor tissues, whereas anti‐PD‐L1 did not affect infiltration. Interestingly, pH‐triggered release of anti‐PD‐L1 enhanced antitumor activity by alleviating local immune suppression. In vivo studies using A20 (lymphoma) and CT26‐CEA (colorectal carcinoma) tumor models demonstrated the improved efficacy of H‐P@CAR‐T. Hyaluronidase‐conjugated CAR‐T cells exhibited deeper tumor infiltration and reduced perivascular retention.^45^ Although short‐term stability of cell‐surface hyaluronidase and enhanced tumor accumulation of engineered cells were demonstrated, the in vivo pharmacokinetics of the conjugated enzyme remain to be characterized. Collectively, H‐P@CAR‐T cells achieved superior tumor growth inhibition and prolonged survival without overt systemic toxicity, highlighting the advantage of combining ECM‐targeting enzymes and immunotherapeutics.^45^

Building on CAR‐T‐based strategies, later studies developed extracellular vesicle‐based platforms whose nanoscale size and tumor‐retentive behavior enabled localized and efficient hyaluronidase delivery.^46^, ^47^ Beyond physical ECM degradation, hyaluronidase activity may also engage immune‐stimulatory pathways. In small extracellular vesicles (sEVs) derived from PH20‐expressing HEK293T cells (sEV‐PH20), degradation products of high‐molecular‐weight HA activated CD103^+^ dendritic cells (DCs) via Toll‐like receptor 4 (TLR4) signaling, thereby enhancing CD8^+^ T cell responses (Figure 1c). In murine melanoma (B16F10‐Ova) and breast cancer (4T1) models, sEV‐PH20 increased CD8^+^ T cell infiltration and suppressed tumor growth. Upon co‐administration of sEV‐PH20 and anti‐PD‐L1 in tumor‐bearing mice, antitumor immunity and durable tumor suppression were further amplified. Additionally, combination therapy with sEV‐PH20 and anti‐PD‐L1 meaningfully suppressed tumor growth and enhanced overall survival in an autochthonous tumor model using MMTV‐PyMT mice. Hyaluronidase‐mediated ECM remodeling may convert immunologically “cold” tumors into “hot” tumors, thereby enhancing responsiveness to immune‐checkpoint inhibitors, which typically exhibit resistance in cold tumors.^47^

To further enhance the tumor selectivity of hyaluronidase, researchers designed a pH‐ and matrix metalloproteinase‐2 (MMP‐2) dual‐responsive polymer/calcium phosphate (CaP) hybrid platform. This nanocarrier enabled the simultaneous delivery of hyaluronidase, interleukin‐12 (IL‐12), and the anti‐PD‐L1 antibody into tumors. To ensure tumor‐specific accumulation and reduce off‐target effects, anti‐PD‐L1 was shielded with PEG‐containing structures. The acidic TME triggered the release of hyaluronidase and IL‐12 from the pH‐sensitive CaP nanocarrier and simultaneously induced PEG de‐shielding via cleavage of acid‐labile bonds. Hyaluronidase facilitated ECM degradation and immune cell infiltration, while IL‐12 strengthened antitumor immunity by promoting CD8^+^ T cell proliferation, cytotoxic activity, and M1 macrophage polarization. In parallel, de‐shielded anti‐PD‐L1 was released from the nanocarrier upon cleavage by MMP‐2, which is abundant in the TME, thereby reversing local T cell immunosuppression. In subcutaneous Hepa1‐6 tumor models, this sequential and stimulus‐responsive delivery strategy translated into superior tumor control and prolonged survival compared with control treatments. Consistent with effective stromal remodeling, treatment reduced intratumoral collagen deposition, likely secondary to alleviation of hypoxia following HA degradation. Importantly, this ECM remodeling and HA depletion did not induce metastasis, as no lung metastases were observed in the hyaluronidase‐treated groups.^48^

The effects of hyaluronidase extend beyond just cleansing the path for CAR‐T cells and monoclonal antibodies. Research has shown that combining intratumoral hyaluronidase injection with a nanovaccine significantly enhanced the performance of the cancer vaccine. The nanovaccine (P/C/O), formulated with polyethylenimine, unmethylated cytosine‐phosphate‐guanine, and ovalbumin (OVA), effectively stimulated DC activation and antigen presentation.^49^ Cancer vaccine immunotherapy aims to generate effector T cells, such as CD8^+^ T cells, that are capable of orchestrating effective antitumor responses. However, to achieve maximal efficacy, immune cells must effectively traffic through the formidable ECM barrier surrounding tumor cells.^50^, ^51^ To further enhance vaccine‐generated T cell infiltration, hyaluronidase has been used to degrade HA within the ECM, resulting in markedly improved CD8^+^ T cell infiltration in groups that received the combined hyaluronidase and P/C/O regimen. Consistent with enhanced immune infiltration and activation, combination treatment significantly increased levels of interferon gamma (IFN‐γ) and tumor necrosis factor alpha (TNF‐α). This ECM remodeling‐assisted vaccination strategy led to significant tumor suppression in B16‐OVA melanoma models.^49^

## 4 COLLAGENASE

Collagens, one of the most abundant components of the ECM, accumulate at high densities and undergo extensive remodeling during cancer progression. These fibrous proteins influence several aspects of the TME by shaping physical barriers, altering tissue mechanics, and impairing immune function, which in turn compromises the efficacy of therapeutic interventions.^52^, ^53^ In particular, collagen not only affects immune cell migration but can also suppress their antitumor activity by binding to specific receptors.^54^, ^55^ Indeed, collagen‐mediated immunosuppression may also compromise immunotherapeutic outcomes, as reported by diminished responses to anti‐programmed cell death protein 1 (PD‐1)/PD‐L1 therapies in high‐collagen lung tumors, as well as negative impacts on CAR‐T cells.^56^, ^57^ In parallel with its immunosuppressive effects, abnormal collagen accumulation also contributes to adverse alterations in the hydraulic properties of tumor tissue. Predictably, abnormal elevation of interstitial fluid pressure (IFP) can reduce transvascular and interstitial convection in tumors, thereby preventing therapeutics, particularly macromolecules, from effectively reaching their targets.^58^ Early efforts to overcome this barrier involved systemic administration of collagenase. In osteosarcoma xenografts, following intravenous administration of collagenase, the uptake of the osteosarcoma‐associated monoclonal antibody (TP‐3) increased by approximately twofold, and its tissue distribution was improved.^11^ However, the lack of specificity and potential off‐target effects associated with systemic collagenase administration have limited its translational applicability. Consequently, more recent strategies have focused on developing localized and responsive collagenase delivery systems to enhance precision and therapeutic efficacy within the tumor microenvironment. One such approach has been explored in pancreatic ductal adenocarcinoma (PDAC), where abnormal collagen accumulation constitutes a significant barrier to therapeutic access.^59^ Specifically, oncogenic type I collagen (Col1) homotrimers (α1/α1/α1), produced explicitly by PDAC cancer cells, promote oncogenic signaling and tumor growth through α3β1 integrin expressed on cancer cells.^60^ Notably, this oncogenic Col1 differs from the well‐known Col1 heterotrimer (α1/α2/α1), which is produced by cancer‐associated fibroblasts (CAFs).^60^ However, because collagenase lacks intrinsic selectivity and may degrade both oncogenic and normal ECM collagen, its therapeutic benefit depends on restricting enzymatic activity in proximity to PDAC cells, where oncogenic Col1 is present at high levels. To overcome poor parenchymal penetration, motile, facultative anaerobic *Escherichia coli* Nissle 1917 (EcN) was used as a tumor‐targeting carrier, taking advantage of the preferential accumulation of bacteria in hypoxic tumor regions.^59^, ^61^ Collagenase and anti‐PD‐L1 were formulated into reactive oxygen species (ROS)‐responsive protein nanocages and conjugated to the surface of EcN (Col/anti‐PD‐L1@EcN), enabling ROS‐triggered local release and degradation of oncogenic collagen. In the orthotopic PDAC model, Col/anti‐PD‐L1@EcN locally reduced oncogenic Col1 and attenuated downstream integrin α3β1‐focal adhesion kinase (FAK) signaling (Figure 1d). Concomitantly, treatment decreased immunosuppression, increased CD8^+^ T cell infiltration, and enhanced immune‐checkpoint blockade efficacy. Moreover, Col/anti‐PD‐L1@EcN extended survival in orthotopic PDAC‐bearing mice and also demonstrated broader efficacy by suppressing primary tumor growth and reducing lung metastatic burden in a 4T1 metastatic breast cancer model.^59^

In line with efforts to minimize off‐target collagen degradation, two EcN strains were engineered to release bacterial collagenase (EcN‐ColG) and an immunotoxin (EcN‐CDPE) within the TME. CDPE is a recombinant fusion protein composed of an anti‐CD47 nanobody (CD47nb), which was fused to a truncated *Pseudomonas* exotoxin A (PE38KDEL). The toxin induces tumor cell death by inhibiting protein synthesis.^62^ Notably, the targeting domain of the CDPE not only enabled the engineered immunotoxin to kill tumor cells in a CD47nb‐dependent manner, but also enhanced phagocytic activity by disrupting the CD47‐SIRPα interaction. Each engineered probiotic released its respective payload in a lysis‐dependent manner upon induction with L‐arabinose. After systemic administration, both engineered probiotics preferentially colonized the tumor site, where the release of ColG significantly reduced intratumoral collagen. This facilitated deeper CDPE penetration, suppressed tumor proliferation, and elevated intratumoral IFN‐γ levels in the 4T1 animal tumor model.^63^

Another strategy to overcome the physical barrier of the tumor involved the co‐delivery of trastuzumab and collagenase using a thermosensitive in situ gel‐forming formulation. Peritumoral injection of a biodegradable poly(lactic‐co‐glycolic acid) (PLGA)‐PEG‐PLGA polymer containing collagenase and trastuzumab (Col/Tra/Gel) initiated the degradation of intratumoral collagen, resulting in improved therapeutic penetration and suppression of tumor growth in HER2^+^ tumor‐bearing mice. Furthermore, tumors treated with Col/Tra/Gel showed higher levels of apoptosis and more substantial anti‐vasculature effects than other groups. Importantly, Col/Tra/Gel sustained protein release, potentially mitigating the rapid in vivo clearance of free collagenase.^64^

While targeted delivery strategies have shown promise in enhancing therapeutic penetration, recent efforts have advanced this approach by engineering anti‐MSLN‐CAR‐T cells equipped with cellular backpacks using DV1‐decorated collagenase nanogels (DV1@O‐Colase#CAR‐T). Given the short in vivo half‐life of collagenase, the nanogel was designed to protect its enzymatic activity while enabling targeted delivery. These nanogels (DV1@O‐Colase) were synthesized by cross‐linking collagenase with oxidized sodium alginate, followed by surface modification with DV1 peptide. This strategy aimed not only to degrade the physical ECM barrier but also to enhance immune cell navigation within the TME.^65^ Apart from the oncogenic collagen discussed previously, CAFs are also key contributors to the collagen‐rich ECM that hinders immune cell trafficking.^66^ The studies revealed that physical obstruction is not the only confounding factor. Indeed, aberrant secretion of chemokine (C‐X‐C motif) ligand 12 (CXCL12) by CAFs can take a drastic toll on TME.^67^ The interaction of chemokine receptor 4 (CXCR4) on T cells with CXCL12 traps T cells within the CXCL12‐rich region surrounding CAFs in the ECM, resulting in inefficient tumor eradication.^68^, ^69^ Therefore, functionalization with the DV1 peptide, a CXCR4 antagonist, not only stably anchored the nanogel backpack to CAR‐T cells via receptor‐ligand interactions but also disrupted CXCR4/CXCL12‐mediated trapping, enabling effective T cell navigation into tumor islets.^65^ Notably, CXCR4 is also expressed on pancreatic cancer cells, making them prone to metastasis; accordingly, the DV1‐decorated nanogels helped impede tumor spread in treated groups.^65^, ^70^, ^71^ Armed CAR‐T cells with DV1@O‐Colase nanogels showed enhanced cell motility by combining ECM degradation with CXCR4 inhibition. In an orthotopic pancreatic cancer model using the Panc02 cell line, DV1@O‐Colase nanogels accumulated at the tumor site, enhanced CD8^+^ T cell infiltration and localization, and significantly suppressed tumor growth. In contrast, administration of unformulated collagenase resulted in no meaningful outcome in the study, underscoring the necessity of targeted delivery. To further assess the efficacy, AsPC‐1 tumor‐bearing mice were treated with backpack‐equipped CAR‐T cells. Due to collagen degradation and enhanced localization throughout the tumor, DV1@O‐Colase#CAR‐T cells significantly outperformed other groups, achieving complete tumor regression in some mice and extending their survival. Interestingly, co‐administration of DV1@O‐Colase with anti‐PD‐1 also improved antitumor responses compared to anti‐PD‐1 alone, which may be attributed to increased CD8^+^ T cell infiltration. Moreover, the adjunct therapy induced a notable increase in granzyme B expression in CD8^+^ T cells and IFN‐γ expression in CD4^+^ T cells. Collectively, this combinatorial strategy also alleviated the hostile tumor microenvironment, as evidenced by a significant reduction in the proportions of T_reg_ cells and myeloid‐derived suppressor cells.^65^

Given the various attempts to rationally target collagen in the TME, a critical question must be addressed before pursuing such interventions: when does collagen depletion help, and when might it be harmful? To answer this, collagen‐targeting strategies should be guided by a context‐dependent understanding of collagen's role within the TME, rather than by viewing collagen as a uniformly harmful target. Although remodeling dense collagen can restore immune‐cell access and enhance responsiveness to immunotherapy, paradoxical effects have also been reported, as indiscriminate stromal depletion may increase tumor aggressiveness and immunosuppression and alter tumor vascularity.^72^ In collagen‐rich lung tumors, dense stromal matrix restricted T cell access to tumor nests, whereas collagen reduction improved T cell infiltration and responsiveness to immune‐checkpoint blockade.^54^ In contrast, studies in PDAC showed that depletion of alpha‐smooth muscle actin (αSMA^+^) myofibroblasts, or myofibroblast‐derived Col1, accelerated tumor progression, enhanced immunosuppression, altered vascularity or hypoxia, and reduced survival.^73^, ^74^ Collagen remodeling is most likely to be beneficial when collagen represents a dominant physical barrier in tumors containing pre‐existing but spatially excluded immune cells or compressed, poorly perfused vessels. Conversely, it may be harmful when collagen‐producing stromal programs restrain tumor spread or angiogenesis. Importantly, current collagenase‐based approaches are generally not designed to distinguish collagen by cellular origin and may therefore degrade both tumor‐promoting, cancer cell‐derived collagen and tumor‐restraining, CAF‐derived collagen. Therefore, to maximize the therapeutic benefit of collagenase, patient stratification should consider tumor type, baseline immune localization, and the source and structural composition of collagen.

## 5 NATTOKINASE

Most studies have focused on specific ECM components; however, emerging evidence suggests that other ECM proteins also play essential roles in tumor fortification. Among these, fibronectin has emerged as a crucial stromal component contributing to therapeutic resistance.^75^ Fibronectin is an ECM protein with diverse functions that is abundantly produced by CAFs. The abnormally accumulated fibronectin can lead to resistance to cancer therapeutics by reinforcing physical barriers within the tumor microenvironment.^75^ To this end, fibronectin in the cancer‐affected ECM was targeted by nattokinase, and studies have investigated whether its degradation could enhance tumor responsiveness to therapeutics.^76^ Nattokinase is a 275 amino acid serine protease (~28 kDa) produced by *Bacillus subtilis* during the fermentation of soybeans to produce natto, a traditional Japanese fermented food.^77^ The enzyme is best known for its potent fibrinolytic activity, which underlies the cardiovascular benefits associated with natto consumption.^78^ Owing to its fibrinolytic activity and capacity to remodel ECM architecture, nattokinase has been repurposed for cancer‐related applications. Intratumoral administration of this enzyme remodeled the tumor ECM by degrading fibronectin, which improved tissue perfusion, reduced hypoxia, and indirectly suppressed CAFs activity (Figure 1e). Taken together, these changes enhanced therapeutic accessibility within the tumor microenvironment. As a result, nattokinase treatment enhanced the efficacy of both chemotherapy and radiotherapy in preclinical tumor models. Remarkably, nattokinase pretreatment also facilitated CAR‐T cell infiltration, resulting in a sixfold increase in intratumoral CAR‐T cell accumulation and superior therapeutic outcomes in breast tumor xenografts.^76^ However, in the study discussed above, nattokinase was administered intratumorally, and its systemic pharmacokinetics and long‐term safety remain insufficiently characterized.^76^ Moreover, as nattokinase is a bacterially derived protein with documented allergenic potential,^79^ its immunogenicity following repeated parenteral administration requires careful evaluation. These safety considerations will need to be addressed before clinical translation.

## 6 SIALIDASE

Under physiological conditions, sialic acid plays important roles; however, hypersialylation in diseased conditions mediates tumor cell progression and correlates with a poor prognosis for patients with cancer.^80^ Excessive sialylation forms a protective cloak that enables tumor cells to evade immune surveillance. This immune‐evasive effect can arise through more than one mechanism. Dense cell‐surface sialylation can limit access of the NK cell activating natural killer group 2D (NKG2D) to its ligands, thereby weakening activating signals from tumor cells.^81^ Sialoglycans can also engage inhibitory sialic acid‐binding immunoglobulin‐like lectin (Siglec) receptors on immune cells, which can further reduce NK cell‐mediated cytotoxicity and ADCC.^82^, ^83^, ^84^ Beyond immune evasion, tumor‐cell sialylation can alter ECM interactions and intracellular signaling. In ovarian cancer cells, sialylation of β1 integrin increased adhesion to and migration on collagen I, potentially facilitating tumor invasion.^85^ In addition, hypersialylated fibroblast growth factor receptor 1 (FGFR1) may alter tumor‐intrinsic signaling, thereby enhancing tumor cell migration and resistance to conventional chemotherapeutics.^86^ Based on the known impact of hypersialylation on cancer progression, various attempts have been made to target sialic acid‐shielded tumor cells with an enzyme. Despite encouraging preclinical and early clinical findings, several translational limitations remain.^87^, ^88^ First, sialic acids are found on many cells, and off‐target effects may cause significant adverse reactions. Monitoring for such effects would benefit from tracking hematologic and immune‐cell desialylation and cytokine release, alongside anti‐drug antibody (ADA) development, which has already been shown to limit repeated dosing of at least one construct in preclinical studies.^89^ Besides blood cells, endothelial cells may also be susceptible to off‐target desialylation. Sialic acids within the endothelial glycocalyx can limit access to adhesion molecules; their removal by untargeted sialidase can disrupt glycocalyx integrity and enhance tumor‐cell attachment to the endothelium, raising a potential concern for metastatic seeding.^90^ However, whether tumor‐targeted sialidase constructs produce comparable endothelial effects remains unknown. Thus, more recent attention has focused on leveraging bioengineering approaches to improve targeted delivery and reduce immunogenicity. Another limitation is the limited durability of desialylation, as cell‐surface sialylation may recover after sialidase treatment; early clinical data suggest tumor resialylation following enzyme clearance,^88^ although recovery kinetics remain poorly studied across different tumor types. Therefore, tumor assessment at different time points may be needed to determine the durability of desialylation. In this regard, a lectin‐staining pattern consistent with reduced tumor sialylation after treatment would support local desialylation, while decreased binding of probes that recognize Siglec‐engaging sialoglycans would indicate a reduction in these immunosuppressive ligands.^14^, ^91^, ^92^

Initial attempts to address the challenges discussed above used an antibody‐sialidase conjugate. The researchers demonstrated that *Vibrio cholerae* sialidase, selected for its ability to cleave various sialic acid linkages, made breast cancer cells more susceptible to ADCC in the presence of trastuzumab. To minimize the off‐target effect of the enzyme, trastuzumab was chemically fused to sialidase (T‐Sia) without interfering with its ability to bind to FcγRIII (CD16) on NK cells. Notably, T‐Sia targeted sialic acids in a HER2‐dependent manner, although some nonspecific enzymatic activity was observed, especially at high doses. By removing tumor‐cell surface sialic acids, targeted desialylation can increase the accessibility of NKG2D ligands, thereby favoring activating interactions between NK cells and tumor cells. Interestingly, this antibody‐enzyme conjugate has the potential to keep trastuzumab at the forefront, as it enhances the therapeutic response in cells with low HER2 and high Siglec ligand expression. In contrast, tumor cells with high HER2 levels can deliver a strong activating signal through FcγRIII, despite Siglec‐mediated inhibition.^91^

Additional studies further refined this strategy by chemically fusing the *Salmonella* Typhimurium sialidase NanH to trastuzumab (T‐Sia 2). By replacing *V. cholerae* sialidase with *S*. Typhimurium sialidase, T‐Sia 2 showed reduced off‐target desialylation, while still promoting NK‐cell‐mediated ADCC of cells with moderate to low HER2 expression.^14^ To enhance the stability of the conjugate, the hydrolysis‐prone oxime bond used in the original T‐Sia, based on *V. cholerae* sialidase, was replaced with a more stable linkage formed via hydrazino‐iso‐Pictet‐Spengler (HIPS) chemistry.^14^, ^91^ Treatment of the trastuzumab‐resistant EMT6‐HER2^+^ orthotopic breast cancer model with T‐Sia 2 significantly delayed tumor growth and prolonged the survival of the treated group. Despite the promising selectivity of the designed therapeutic, off‐target desialylation is still observed in tissues at higher T‐Sia 2 doses. In addition to an enhanced CD8^+^ T cell/T_reg_ ratio, T‐Sia 2‐treated groups revealed higher numbers of activated (CD69^+^) and cytotoxic (granzyme B^+^) CD8^+^ T cells and NK cells. Mechanistic studies revealed that the therapeutic effect of T‐Sia 2 was not solely dependent on ADCC but also involved modulation of the immune system through the Siglec axis. Specifically, T‐Sia 2 reduced the engagement of Siglec‐E, the murine functional homolog of human Siglec‐9, with its ligands, thereby relieving inhibitory signaling and enhancing antitumor responses.^14^ Subsequent investigations further revealed that T‐Sia 2 could also repolarize tumor‐associated macrophages toward the M1 phenotype, which exhibited antitumor activity.^93^

Motivated by the evidence of reciprocal modulation between PD‐L1 and sialic acid pathways, an anti‐PD‐L1 nanobody (Nb16) was fused to a sialidase from *Actinomyces* sp. (Nb16‐Sia).^94^ In terms of therapeutic delivery, nanobodies exhibit better tumor permeation compared to conventional monoclonal antibodies.^95^ Moreover, the sialidase from an oral commensal bacterium shared structural similarity with human Neu3, which may help reduce undesired immune responses. Nb16‐Sia induced dose‐dependent tumor suppression in CT26 colon cancer models and similarly suppressed tumor growth in MC38 tumors.^94^ Following treatment with Nb16‐Sia, frontlines in the TME were reinforced, as evidenced by a notable increase in NK cell infiltration, enhanced antigen‐presenting capability of macrophages, and improved T cell functionality. Notably, the nanobody‐enzyme conjugate not only inhibited M2 polarization from M0 macrophages but also repolarized existing M2 cells toward the M1 phenotype. The C‐type lectin signaling pathway might regulate this process.^94^

Complementary to PD‐L1‐directed targeting, a recent immune‐cell‐directed strategy conjugated *S*. Typhimurium sialidase to an anti‐PD‐1 IgG4 antibody, thereby concentrating enzymatic activity on PD‐1‐expressing lymphocytes.^89^ This design is mechanistically relevant because the efficacy of PD‐1 blockade depends on CD28‐B7 co‐stimulation between T cells and antigen‐presenting cells, which may be impaired by cell‐surface sialylation.^89^, ^96^ Following enzymatic conjugation, the anti‐PD‐1‐sialidase construct retained both PD‐1 recognition and catalytic activity and preferentially desialylated PD‐1‐positive cells. Targeted desialylation enhanced T cell effector function and improved tumor‐cell killing by exhausted tumor‐reactive T cells.^89^ Given that the wild‐type conjugate caused off‐target desialylation, an attenuated conjugate incorporating the active‐site R309A variant was developed. In the B16‐OVA melanoma model with adoptively transferred OT‐I cells, this construct improved tumor control and survival. It was associated with M1‐like macrophage polarization, reduced terminally exhausted CD8^+^ T cell populations, and enrichment of progenitor‐exhausted CD8^+^ T cells. From a delivery perspective, conjugation of sialidase to anti‐PD‐1 produced greater desialylation and T cell activation than co‐administration of the unconjugated agents, supporting the value of targeted enzyme delivery. Importantly, the emergence of anti‐drug antibodies limited dosing to three administrations.^89^

Beyond antibody‐ and nanobody‐based conjugates, sialidase has also been incorporated into bispecific T cell engagers (BiTEs). BiTEs, a class of bispecific antibodies, bind a tumor‐associated antigen and CD3 on T cells to promote T cell‐mediated cytotoxicity, whereas checkpoint inhibitory T cell engagers (CiTEs) add an immunomodulatory component to this core.^97^ In one study, *S*. Typhimurium sialidase was chemically conjugated as an immunomodulatory component to a HER2 × CD3 bispecific T cell engager, generating a tri‐protein CiTE. The sialidase‐containing CiTE retained HER2‐ and CD3‐binding as well as enzymatic activity, although CD3 binding was reduced relative to the corresponding BiTE. The CiTE desialylated both HER2‐high and HER2‐low breast cancer cells, although it desialylated HER2‐high cells at lower concentrations. It also enhanced T cell‐mediated killing of HER2‐positive MDA‐MB‐231 cells in vitro and showed greater activity at lower concentrations than both the anti‐PD‐1 CiTE and the core HER2 × CD3 BiTE.^98^

A subsequent study advanced this strategy by genetically fusing *Bifidobacterium longum* subsp. *infantis* sialidase to either terminus of a HER2 × CD3 BiTE. Although both constructs desialylated HER2‐positive cells, the C‐terminal fusion showed greater desialylation and T cell‐mediated cytotoxicity.^16^ The two studies indicate that construct architecture, including component arrangement and fusion orientation, may influence biological activity.^16^, ^98^ The optimized fusion preferentially desialylated HER2‐positive cells while showing minimal activity toward HER2‐negative cells in mixed cultures, and its relative enhancement of cytotoxicity was greater in HER2‐low breast cancer cell lines. In the tested in vitro model, mechanistic experiments suggested that this improvement was associated primarily with stronger immunological synapse formation rather than Siglec‐mediated checkpoint inhibition. Sialidase was also fused to CD19‐ and prostate‐specific membrane antigen (PSMA)‐targeting BiTEs, which enhanced T cell activation and tumor‐cell killing in vitro. In different animal models, HER2‐ and CD19‐targeting fusions improved tumor control in breast cancer and leukemia xenografts, respectively. An EGFR‐targeting fusion also delayed tumor growth and prolonged survival in an immunocompetent melanoma model. Removing CD8^+^ T cells eliminated the treatment‐associated advantage in tumor control, supporting a key role for these cells in this model. Immune profiling further revealed a shift toward greater representation of intratumoral CD8^+^ T cells and NK cells, together with reduced representation of myeloid cells.^16^ However, the platform remains preclinical, and its biodistribution, off‐target desialylation, and safety under repeated systemic dosing require further evaluation.

Building on the promising results of antibody‐mediated sialidase targeting, efforts have begun to explore further the effect of sialidase on the therapeutic outcome of engineered cells. In this setting, CAR‐T cells targeting EGFR or CD19 were engineered to secrete *Clostridium perfringens* neuraminidase (*CpNA*). *CpNA* secretion enhanced the cytotoxic activity of CAR‐T cells in vitro and increased T cell‐tumor cell interactions. Importantly, this strategy reduced CAR‐T cell differentiation during ex vivo expansion, resulting in a more naïve‐like phenotype (CCR7^+^; CD45RO^−^), associated with superior in vivo persistence and tumor control. Notably, *CpNA* secretion from engineered CAR‐T cells increased in an antigen‐responsive manner, which may help restrict enzyme exposure to sites of antigen engagement. Across U87 glioblastoma, Nalm6 leukemia, and B16F10 melanoma models, *CpNA*‐secreting CAR‐T cells produced greater antitumor effects than the respective controls, while the Nalm6 rechallenge model additionally showed substantially lower tumor burden and longer survival.^99^ Complementary to the secretion fashion, recent work has localized *S*. Typhimurium sialidase activity by tethering the enzyme to NK cells through a modular coupling strategy, enhancing cytotoxicity across diverse tumor models; this approach is discussed further in the mucinase section.^100^

As part of ongoing efforts to enhance the therapeutic performance of cell‐based immunotherapies through enzymatic modulation, induced pluripotent stem cell‐derived chimeric antigen receptor‐macrophage (CAR‐iMac) expressing anti‐MSLN‐CAR was evaluated in combination with sialidase.^101^ In addition to ADCC, antibody‐dependent cellular phagocytosis (ADCP) is a major effector function in antitumor responses and contributes meaningfully to innate immune surveillance of tumor cells. However, tumor‐associated sialoglycans may suppress macrophage‐mediated tumor‐cell clearance by engaging inhibitory Siglecs on macrophages.^102^, ^103^ In this context, pretreatment of ovarian cancer cells with sialidase enhanced CAR‐iMac‐mediated tumor killing by improving phagocytosis, promoting phagosome formation, and boosting CAR‐iMac polarization in vitro, accompanied by increased secretion of pro‐inflammatory cytokines such as TNF‐α, interleukin‐1 beta (IL‐1β), and IFN‐γ. Because free sialidase lacks intrinsic tumor specificity, novel targeted desialylation strategies were subsequently developed by conjugating sialidase to a non‐small cell lung cancer‐targeting peptide (TP‐Sia) or folate (Fa‐Sia), enabling localized activity in the Lewis lung carcinoma and orthotopic ovarian cancer mouse models, respectively. Although this strategy reduced sialic acid levels within the TME, the enzyme alone did not show significant antitumor activity. Animal studies confirmed that combining sialidase with CAR‐iMacs or bone marrow‐derived macrophages (BMDMs) reduced tumor growth. Targeted sialidase treatment also enhanced NK cell and M1‐macrophage infiltration. Sialidase activity reduced the ligands for the inhibitory receptors Siglec‐5 and Siglec‐10, making tumor cells more vulnerable to CAR‐iMacs. Surprisingly, a similar effect was observed when Siglec‐5 and Siglec‐10 were knocked out in CAR‐iMacs.^101^ The mechanism of action of sialidase is summarized in Figure 2.

**FIGURE 2:**
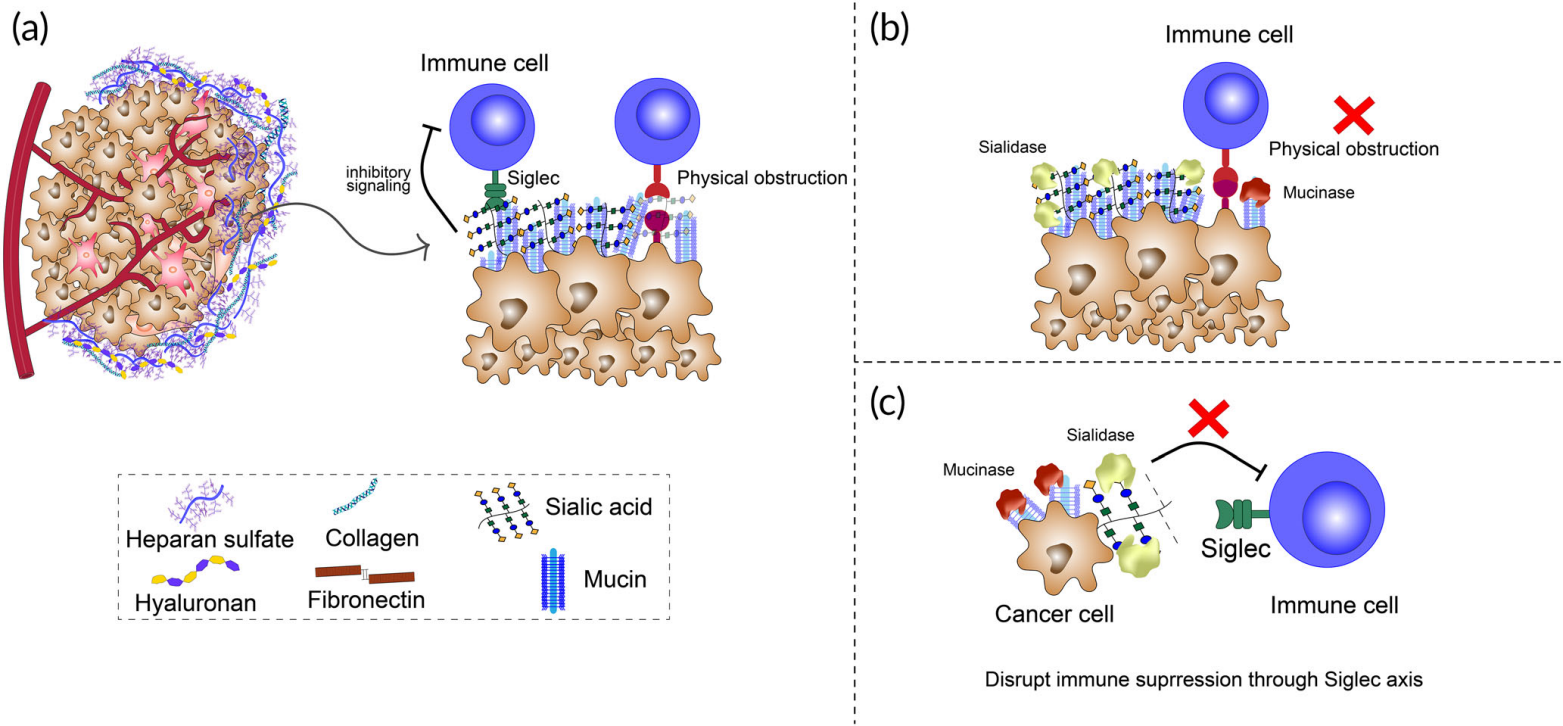
Enzymatic cleavage of the tumor‐associated glycocalyx: (a) excess mucins and hypersialylated glycans surrounding cancer cells can impede immune‐cell engagement by physically blocking receptor‐ligand interactions and promoting Siglec‐mediated immunosuppression; (b) sialidase and mucinase reduce physical shielding by cleaving glycocalyx components; (c) substrate cleavage also depletes Siglec ligands, thereby disrupting inhibitory Siglec signaling.

In addition to promising preclinical results, newer glycocalyx‐targeting approaches such as E‐602, a first‐in‐class bi‐sialidase (engineered human sialidase Neu2‐IgG1 Fc fusion protein), are showing early clinical promise. By cleaving sialic acids from tumor and immune‐cell sialoglycans, E‐602 disrupts the Siglec axis and promotes immune activation. In the Phase 1/2 GLIMMER‐01 study, E‐602 demonstrated tumor desialylation and early clinical activity, particularly in hypersialylated tumors, when combined with cemiplimab (anti‐PD‐1).^88^ More recently, E‐612, a modified form of E‐602 with improved stability and manufacturability, showed preclinical activity in prostate cancer bone metastasis. Treatment reduced Siglec‐7/9‐engaging sialoglycans, suppressed metastatic growth, and prolonged survival, while desialylation of circulating white blood cells indicated systemic enzymatic activity.^92^ Using human sialidases may reduce concerns regarding immunogenicity; however, untargeted enzymatic activity remains a challenge, as it may increase the likelihood of off‐target effects. E‐688 (HLX316) is an engineered human sialidase fused to a B7‐H3‐targeting nanobody. In preclinical studies, E‐688 produced deeper and more durable tumor desialylation than untargeted sialidase and enhanced both ADCC and ADCP. It was also well tolerated in a repeat‐dose non‐human primate toxicology study.^104^

## 7 MUCINASE

Mucins are characterized by tandem repeat regions rich in proline, threonine, and serine, which serve as dense sites for O‐glycosylation. These structural features confer extended, heavily glycosylated domains that contribute to mucin barrier function.^8^, ^105^ Mucins play pivotal roles in cancer progression by altering cell adhesion, promoting immune evasion, and forming protective biophysical barriers. Their overexpression, particularly MUC1, is common in carcinomas and correlates with poor prognosis.^106^ A recent study demonstrated that mucin‐rich glycocalyx thickness inversely correlates with immune cell‐mediated cytotoxicity, with changes of approximately 10 nm altering cancer‐cell susceptibility to immune cell attack. Notably, mucin‐associated sialoglycans can engage inhibitory Siglec receptors; however, mucin‐mediated protection was not strictly dependent on Siglec signaling.^100^

To test whether enzymatic barrier removal could restore immune killing, researchers used StcE, a mucin‐selective metalloprotease derived from *E. coli* O157:H7, to cleave mucin ectodomains at the tumor cell surface. StcE treatment reduced glycocalyx thickness and restored NK‐cell‐mediated killing in both engineered mucin‐expressing epithelial models and mucin‐high breast cancer cells. Translationally, two immunoengineering approaches were developed to localize StcE delivery on NK cells, promoting targeted glycocalyx disruption at the NK‐tumor cell interface (Figure 2). First, leveraging lectin‐mediated tethering of StcE to NK cells, anti‐HER2 CAR‐NK cells armed with surface‐tethered StcE exhibited enhanced cytotoxicity against mucin‐rich cancer cells.^100^ To improve therapeutic versatility, the authors developed a modular leucine‐zipper “Zip‐NK” platform. In this system, NK cells expressing a surface RR zipper are loaded with EE‐tagged enzymes, enabling high‐affinity and controllable surface display of StcE and other enzymes (e.g., sialidase) without relying on native glycan binding.^100^ This modular design enables interchangeable enzyme loading,^100^ which may offer a path toward more standardized cell‐product manufacturing.

In addition to arming cell‐based immunotherapies, mucinase‐based strategies can also improve antibody‐mediated targeting by reducing steric shielding within the mucin‐rich glycocalyx. However, systemic exposure to wild‐type StcE raises safety concerns, as high‐dose administration in mice caused platelet depletion and hemorrhagic toxicity. Accordingly, StcE was advanced through protein engineering to generate an attenuated variant (eStcE) that retained specificity for mucin glycopeptide motifs while minimizing off‐target effects.^13^ For targeted delivery, eStcE was genetically fused to a nanobody targeting the HER2 receptor, creating anti‐HER2‐eStcE. The fusion protein maintained mucinase activity and selectively degraded mucins on HER2^+^ cancer cells while leaving HER2^−^ cells unaffected, as observed in animal studies. Therapeutic efficacy of anti‐HER2‐eStcE was demonstrated in two complementary mammary tumor models, in which treatment reduced tumor burden and metastatic spread. Immune profiling further indicated remodeling of the tumor microenvironment in anti‐HER2‐eStcE‐treated groups, with increased conventional DC infiltration, higher granzyme B levels in these cells, and reduced PD‐1 expression on Ly6G^+^ cells.^13^

A thick mucin‐rich glycocalyx can also compromise checkpoint blockade by limiting therapeutic access to cell‐surface checkpoint proteins. To overcome this barrier, StcE was recently incorporated into engineered cell‐membrane fusion nanovesicles (FNVs) that co‐displayed a CD47‐targeting nanobody (nCD47). To reduce off‐target activity, the design combined deletion of the nonspecific‐binding domain of StcE with nCD47‐mediated targeting.^107^ Conceptually similar to the leucine‐zipper‐based modular strategy described earlier,^100^ SpyTag‐SpyCatcher enabled the controlled covalent display of StcE on nanovesicles.^107^ This strategy mitigates the limitations associated with random chemical conjugation or the direct expression of large fusion proteins.^107^ The CD47‐SIRPα axis functions as an innate immune checkpoint that inhibits macrophage‐mediated phagocytosis of tumor cells.^108^ As a result of StcE‐mediated glycocalyx degradation, the checkpoint blocker gained better access to CD47, thereby enhancing CD47‐SIRPα blockade and macrophage phagocytosis.^107^ Following intravenous administration, StcE‐nCD47‐FNVs showed greater tumor persistence than free StcE, which was rapidly cleared and caused thrombocytopenia, periocular inflammation, and hepatotoxicity. In separate in vitro assays, free StcE caused hemolysis and reduced endothelial‐cell viability. In CT26 colorectal tumors and a 4T1 lung‐metastasis model, co‐display of StcE and nCD47 produced stronger tumor control than either modality alone and was associated with M1‐like macrophage polarization and increased CD8^+^ T cell infiltration.^107^ The barrier‐specific enzyme, delivery, immunotherapy‐pairing, and safety considerations discussed across these six extracellular barrier types are summarized in Figure 3.

**FIGURE 3:**
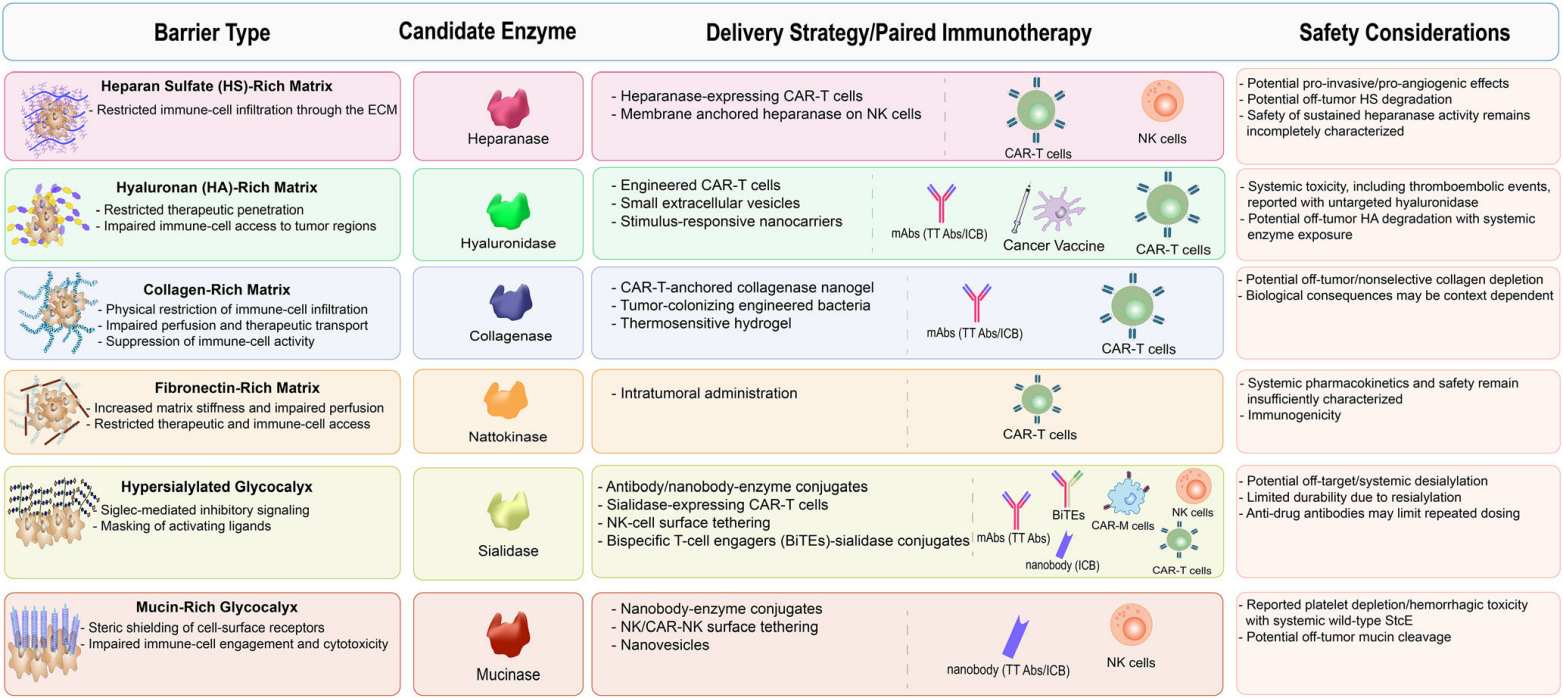
A framework summary of barrier‐specific therapy mapping linking extracellular barrier types to candidate enzymes, delivery strategies, paired immunotherapies, and safety considerations. Pairings reflect experimentally investigated combinations rather than preferred clinical options, and most remain preclinical. CAR‐T cells, chimeric antigen receptor‐T cells; ECM, extracellular matrix; ICB, immune‐checkpoint blockade antibodies; NK, natural killer; TT Abs, tumor‐targeting antibodies.

## 8 TRANSLATIONAL CHALLENGES AND FUTURE DIRECTIONS

For many years, cancer drug discovery focused solely on targeting tumor cells, overlooking the crucial role of other players within the tumor microenvironment, which can ultimately undermine therapeutic efforts. While therapeutic strategies, particularly immunotherapeutics, have shown promising results in ideal settings that exclude TME complexity, their real‐world performance in solid tumors remains limited. The gradually changing perspective on the ECM, from a passive scaffold to a dynamic partner of the immune system within the TME, not only opens avenues for novel therapeutic strategies but also uncovers overlooked opportunities for intervention.

This broader view has prompted the development of several strategies to modify its stromal and extracellular features. Some approaches act at the cellular level by targeting matrix‐producing CAFs. For example, antibodies, vaccines, and engineered T cells directed against CAF‐associated markers have been investigated to this aim.^109^ Other strategies suppress ECM production, cross‐linking, turnover, or mechanotransduction. Transforming growth factor beta (TGF‐β) promotes ECM deposition through several signaling pathways. Therefore, small‐molecule inhibitors and monoclonal antibodies have been developed to block TGF‐β signaling and reduce collagen production and other profibrotic effects.^9^ The renin‐angiotensin system has also been pharmacologically modulated; losartan has been investigated for its ability to reduce collagen and HA deposition and decompress tumor blood vessels.^110^

Beyond suppressing ECM production, therapeutic strategies have targeted ECM‐receptor signaling, including integrins and discoidin domain receptors (DDRs), using antibodies or small molecules. Although these approaches may disrupt ECM‐mediated signaling, the widespread expression and physiological functions of these receptors may limit their therapeutic selectivity.^9^ Additionally, endogenous enzymes involved in ECM organization and turnover have been studied as therapeutic targets. Inhibitors of lysyl oxidase and lysyl oxidase‐like proteins (LOX/LOXL) have been developed to reduce collagen cross‐linking and matrix stiffness, whereas MMP inhibitors have been evaluated to modulate matrix turnover.^111^

Unlike the approaches discussed above, path cleansers catalytically remove pre‐existing extracellular substrates, enabling relatively rapid barrier remodeling and potentially altering signaling. This mode of action distinguishes them from strategies that act upstream by targeting stromal cells, ECM production, or remodeling pathways. Nevertheless, most enzymatic strategies targeting the ECM and glycocalyx remain preclinical, with sialidase‐based approaches showing early clinical activity.^88^ To the best of our knowledge, only a limited number of enzymes have been investigated for their ability to enhance immunotherapy. Tables 1 and 2 summarize these studies, and Table 3 compares the evidence maturity and major translational limitations of the principal enzyme classes.

**TABLE 1:** Summary of preclinical studies combining extracellular matrix (ECM)‐targeting enzymes and immunotherapeutics.

Enzyme	Therapeutic	Cancer type	Data	References
Heparanase	Anti‐GD2‐CAR‐T cells stably express HPSE	Neuroblastoma in vitro and in vivo	Enhanced matrix degradation and T cell infiltration; reduced tumor burden; prolonged survival; no observable off‐tumor toxicity	23
Heparanase	NK‐92^CD3/CD8^/GS3™	Head and neck squamous cell carcinoma in vitro and in vivo	Enhanced infiltration in spheroid models; reduced tumor volume and weight; significantly higher number of engineered NK cells detected in tumors; preserved cytokine secretion and cytotoxic function	24
Hyaluronidase	NK cell + trastuzumab (anti‐HER2) + PEGPH20 NK cell + cetuximab (anti‐EGFR) + PEGPH20	Breast cancer in vitro Ovarian cancer in vitro and in vivo	Enhanced access of NK cells to tumor cells; enzymatic depletion of HA resulted in enhanced in vitro ADCC; increased the antitumor activity upon administration of HA‐degrading enzyme in vivo	36
Hyaluronidase	Anti‐MSLN‐CAR‐T expressing sPH20‐IgG2	Gastric carcinoma in vitro and in vivo	Enhanced transwell migration through HA matrix; improved tumor infiltration of T cells; decreased tumor volume and weight; reduced HA levels in tumor	43
Hyaluronidase	Anti‐GPC3‐CAR‐T co‐express IL‐7 and PH20 (G3CAR‐7×20)	Hepatocellular carcinoma in vitro and in vivo	Enhanced CAR‐T cell matrix invasion and tumor infiltration; improved proliferation and memory phenotype; reduced tumor volume; extended survival	44
Hyaluronidase	Hyaluronidase and anti‐PD‐L1 are chemically conjugated to anti‐CD19‐CAR‐T cell Hyaluronidase and anti‐PD‐L1 are chemically conjugated to anti‐CEA‐CAR‐T cell	Colon cancer in vitro and in vivo Lymphoma in vitro and in vivo	Enhanced ECM penetration and tumor infiltration; alleviated TME immunosuppression; increased cytokine release; decreased exhaustion markers (PD‐1, LAG‐3, TIM‐3); superior tumor control and prolonged survival without systemic toxicity	45
Hyaluronidase	sEV‐PH20 + anti‐PD‐L1 (small extracellular vesicles expressing PH20)	Melanoma in vitro and in vivo Breast cancer in vitro and in vivo	TLR4‐mediated immune activation by HA degradation fragments; increased CD8^+^ T cell infiltration, granzyme B, and IFN‐γ production; reduced tumor volume; enhanced the antitumor effect of PD‐L1 blockade to achieve durable tumor regression and immune memory	47
Hyaluronidase	Hybrid nanocarriers to simultaneously deliver hyaluronidase, IL‐12, and the anti‐PD‐L1 antibody shielded with PEG‐containing structures (P‐anti‐PD‐L1‐CaP@H/I)	Hepatocellular carcinoma in vivo	Depleted HA and reduced collagen deposition; increased CD8^+^ T cell infiltration and IFN‐γ expression; promoted M1 macrophage polarization; suppressed tumor growth and improved survival	48
Hyaluronidase	PEI/CpG/OVA nanovaccine + hyaluronidase	Melanoma in vitro and in vivo	Enhanced DC maturation; increased CD8^+^ and OVA‐specific CD8^+^ T cell infiltration; reduced tumor HA; increased IFN‐γ and TNF‐α; reduced tumor size and weight	49
Collagenase	Murine mAb TP‐3 (IgG 2b) + collagenase	Osteosarcoma in vivo	Improved mAbs uptake and tissue distribution	11
Collagenase	Collagenase and anti‐PD‐L1 conjugated on the surface of EcN	Pancreatic ductal adenocarcinoma in vitro and in vivo Breast cancer in vivo	Depleted oncogenic collagen; inhibited integrin α3β1–FAK /MAPK/AKT signaling; increased CD8^+^ T cell infiltration; enhanced IFN‐γ and TNF‐α levels; reduced tumor volume; improved survival	59
Collagenase	EcN expressing immunotoxin CDPE + EcN expressing collagenase	Breast cancer in vitro and in vivo	Decrease intratumoral collagen; enhanced IFN‐γ expression; prolonged survival	63
Collagenase	Biodegradable PLGA‐PEG‐PLGA polymer containing collagenase and trastuzumab (Col/Tra/Gel)	Breast cancer in vivo	Enhanced trastuzumab tumor retention and efficacy; reduced collagen density; Increased tumor cell apoptosis	64
Collagenase	Anti‐PD‐1 + DV1@O‐Colase DV1@O‐Colase nanogels were anchored by receptor‐ligand interaction to anti‐MSLN‐CAR‐T cells	Pancreatic cancer in vitro and in vivo	Disruption of the CXCR4/CXCL12 axis led to suppressed metastasis and enhanced localization of T cells; enhanced tumor regression and prolonged survival; enhanced immune cell function; reduced suppressor cell populations within the TME	65
Nattokinase	Nattokinase + anti‐CD19‐CAR‐T cells	Breast cancer in vitro and in vivo	Fibronectin degradation; modified stiffness; increased perfusion and alleviated hypoxia; indirect inhibition of CAFs; increased number of infiltrated CAR‐T cells in the tumor	76

Abbreviations: ADCC, antibody‐dependent cell‐mediated cytotoxicity; CAFs, cancer‐associated fibroblasts; CAR, chimeric antigen receptor; CEA, carcinoembryonic antigen; Colase, collagenase; CpG, unmethylated cytosine‐phosphate‐guanine; CXCL12, chemokine (C‐X‐C motif) ligand 12; CXCR4, chemokine receptor 4; DC, dendritic cell; EcN, *Escherichia coli* strain Nissle 1917; HA, hyaluronan; mAb, monoclonal antibody; MAPK/AKT, mitogen‐activated protein kinase/protein kinase B; MSLN, mesothelin; NK, natural killer; OVA, ovalbumin; PEI, polyethylenimine; TME, tumor microenvironment.

**TABLE 2:** Summary of preclinical studies combining glycocalyx‐editing enzymes and immunotherapeutics.

Enzyme	Therapeutic	Cancer type	Data	References
Sialidase	Trastuzumab‐*Vibrio cholerae* sialidase	Breast cancer: in vitro	Enhanced NK cell‐mediated ADCC; reduced Siglec‐7/9 ligand binding; enhanced ligand binding to NKG2D; enhanced ADCC by NK cells; enhanced trastuzumab‐dependent ADCC in HER2‐low cells	91
Sialidase	Trastuzumab‐*Salmonella* Typhimurium sialidase	Breast cancer: in vivo	Enhanced CD8^+^ T and NK cell activation; increased CD8^+^/T_reg_ ratio; tumor desialylation; prolonged survival; effective in trastuzumab‐resistant tumors	14
Sialidase	Anti‐PD‐L1 nanobody‐Sialidase (derived from *Actinomyces* sp.) fusion protein	Colon cancer: in vivo	Enhanced intratumoral CD8^+^ and NK cell infiltration; increased IFN‐γ, TNF‐α, granzyme B secretion; repolarized M2 to M1 macrophages via C‐type lectin pathway	94
Sialidase	Anti‐PD‐1‐*S*. Typhimurium sialidase	Melanoma: in vitro and in vivo	Targeted desialylation of PD‐1‐positive immune cells; enhanced T cell effector function; reduced terminal exhaustion; improved tumor control	89
Sialidase	Anti‐HER2/anti‐CD3 BiTE‐*S*. Typhimurium sialidase	Breast cancer: in vitro	Enhanced T cell‐mediated cytotoxicity; desialylation of both tumor and T cells	98
Sialidase	HER2 BiTE‐*Bifidobacterium longum* subsp. *Infantis* sialidase CD19 BiTE‐*B. longum* subsp. *infantis* sialidase EGFR BiTE‐*B. longum* subsp. *Infantis* sialidase PSMA BiTE‐*B. longum* subsp. *infantis* sialidase	Breast cancer: in vitro and in vivo Leukemia: in vitro and in vivo Melanoma: in vitro and in vivo Ovarian cancer: in vitro Prostate cancer: in vitro	Enhanced immunological synapse formation; increased T cell activation and tumor‐cell cytolysis; delayed tumor growth and prolonged survival	16
Sialidase	Anti‐EGFR‐CAR‐T cells expressing *CpNA* Anti‐CD19‐CAR‐T cells expressing *CpNA* Anti‐GD2‐CAR‐T cells expressing *CpNA*	Glioblastoma: in vitro and in vivo Leukemia: in vitro and in vivo Melanoma: in vivo Neuroblastoma: in vivo	*CpNA* enhanced CAR‐T cell tumor control, CD8^+^ T infiltration, and memory‐like phenotype; improved persistence and survival; no systemic toxicity	99
Sialidase	BMDM + Sialidase‐TP Anti‐MSLN‐CAR‐iMac + Sialidase‐folate	Lewis lung carcinoma: in vitro and in vivo Ovarian cancer: in vitro and in vivo	Increased immune infiltration; enhanced ADCP and ADCC; elevated cytokine levels; prolonged survival when combined with CAR‐iMacs	101
Mucinase	StcE tethered NK‐92 StcE + anti‐HER2‐CAR NK‐92 Zip‐NK‐92 + StcE‐EE	Ovarian cancer: in vitro Breast cancer: in vitro Pancreatic cancer: in vitro	Surface‐tethered StcE locally remodeled the tumor‐cell glycocalyx at the NK‐tumor interface and enhanced NK‐92 cytotoxicity; combining StcE display with HER2 CAR expression further enhanced cytotoxicity across multiple mucin‐rich cancer‐cell models	100
Mucinase	Anti‐HER2 nanobody‐eStcE	Breast cancer: in vitro and in vivo	Targeted improvement of NK cell cytotoxicity against HER2‐positive cells; impede the metastatic outgrowth in an animal model; reduced tumor size and prolonged survival	13
Mucinase	StcE‐displaying fusion nanovesicles co‐displaying CD47 nanobody	Colorectal cancer: in vitro and in vivo Breast cancer: in vitro and in vivo	Enhanced CD47 targeting and macrophage phagocytosis; improved tumor accumulation and tolerability; increased antitumor immunity, tumor control, and survival.	107

Abbreviations: ADCC, antibody‐dependent cell‐mediated cytotoxicity; ADCP, antibody‐dependent cellular phagocytosis; BiTE, bispecific T cell engager; BMDM, bone marrow‐derived macrophage; CAR, Chimeric antigen receptor; CAR‐iMac, induced pluripotent stem cell (iPSC)‐derived CAR macrophages; *CpNA*, *Clostridium perfringens* neuraminidase; EGFR, epidermal growth factor receptor; eStcE, engineered StcE; MSLN, mesothelin; NK, natural killer; NKG2D, natural killer group 2D; PSMA, prostate‐specific membrane antigen; TP, non‐small cell lung cancer‐targeting peptide.

**TABLE 3:** Evidence maturity matrix for extracellular barrier‐editing enzyme classes. Stage 1: In vitro or ex vivo mechanistic evidence; Stage 2: In vivo efficacy evidence from animal models; Stage 3: Early clinical pharmacodynamic, safety, or preliminary efficacy evidence; and Stage 4: Comparative or pivotal clinical outcomes, including negative or unsuccessful clinical studies. A higher evidence stage reflects advancement in evaluation and does not necessarily indicate greater efficacy or safety. Systemic PEGPH20 is therefore included as a Stage 4 cautionary example. The staging system has been devised based on the authors' interpretations.

Enzyme class	Highest evidence stage	Main translational blocker
Heparanase	Stage 2	Unresolved safety and nonimmune stromal effects of heparanase activity
Hyaluronidase	Stage 4: Systemic administration of PEGPH20 clinical failure; Stage 2: Immunotherapy combinations	Systemic exposure and treatment‐related adverse effects
Collagenase	Stage 2	Context‐dependent consequences of nonselective collagen depletion
Nattokinase	Stage 2	Limited systemic pharmacokinetic and safety characterization
Sialidase	Stage 3	Limited durability of desialylation
Mucinase	Stage 2	On‐target, off‐tumor substrate cleavage

### 8.1 Matrix reconstitution and compensatory stromal responses

The durability of treatment and the potential for compensatory responses to enzymatic ECM and glycocalyx editing remain relatively underexplored. Considering the dynamic nature of the extracellular barrier, enzymatic depletion may produce a transient therapeutic window rather than permanently removing the barrier. For instance, HA‐rich pericellular matrices were completely reconstituted within 24 h after removal of PH20 in vitro, whereas PEGPH20‐mediated HA depletion in vivo persisted for more than 72 h before gradually recovering.^33^ A similar pattern has been observed with E‐602, for which early clinical data suggest tumor resialylation following enzyme clearance.^88^ Studies of non‐enzymatic stromal interventions further indicate that perturbation of the tumor stroma can engage compensatory resistance mechanisms. Prolonged pharmacological stromal modulation in PDAC resulted in stromal depletion, but treatment resistance eventually emerged in association with activation of a compensatory signaling pathway.^112^ Moreover, genetic deletion of Col1 in αSMA^+^ myofibroblasts led to increased expression of a tumor‐cell‐derived chemokine, which was associated with recruitment of myeloid‐derived suppressor cells and suppression of CD8^+^ T cells.^73^ Matrix reconstitution and compensatory stromal responses may limit the durability of extracellular barrier editing. However, direct evidence of acquired resistance to ECM‐ or glycocalyx‐editing enzymes remains limited.

### 8.2 Tumor heterogeneity and biomarker‐guided patient selection

As with almost all treatment strategies, patients do not benefit equally from the same therapy. This is particularly true in cancer, and ECM/glycocalyx‐editing approaches are no exception.^113^ Extracellular composition and organization vary across tumor types, patient subgroups, and disease states.^3^ Moreover, substrate abundance alone does not establish that it contributes to therapeutic resistance, nor does its enzymatic depletion necessarily improve treatment response. The context‐dependent effects of collagen, discussed in the collagenase section, illustrate this point.^72^ Similarly, findings from PEGPH20 trials in pancreatic cancer suggest that HA‐high status alone may be insufficient to predict treatment outcome.^39^ Therefore, patient selection may benefit from a holistic assessment that considers substrate abundance alongside other factors, such as immune exclusion and immunosuppression. Biopsy‐based omics and advanced spatial imaging techniques can support patient stratification and selection of appropriate barrier‐editing strategies.^114^, ^115^ Relevant omics‐derived features can include non‐coding RNA (ncRNA) profiles associated with mechanical changes in the tumor microenvironment. Matrix stiffness can alter microRNA (miRNA) and long non‐coding RNA (lncRNA) profiles in both tumor and stromal compartments. Dysregulation of these ncRNAs may in turn contribute to tumor progression, stromal activation, and therapy resistance through modulation of ECM‐remodeling and mechanotransduction pathways.^116^ Differences in mechanosensitive ncRNA profiles may therefore provide additional biomarker information for stratifying patients with highly fibrotic or mechanically altered tumors, although their predictive value and clinically relevant thresholds remain to be established.^116^ Moreover, circulating ECM components and fragments, including HA, collagen‐derived neoepitopes, and remodeling enzymes, may provide complementary, minimally invasive biomarkers for pretreatment assessment and longitudinal monitoring.^117^, ^118^ Circulating or exosomal ncRNA profiles may also reflect stiffness‐associated molecular changes within the tumor microenvironment.^116^ However, these markers should be validated as predictors of response to a specific barrier‐editing strategy rather than as prognostic indicators alone. Their clinical application will also require standardized assays and disease‐specific validation.^119^

### 8.3 Discovery and engineering of new enzyme candidates

Despite being a rich source of inspiration, the microbial realm and other natural resources have not yet been thoroughly screened for this purpose. Only a few studies have explored enzymes from bacteria such as *Clostridium histolyticum*, *B. subtilis*, *V. cholerae*, *S*. Typhimurium, *Actinomyces* sp., *B. longum* subsp. *infantis*, *C. perfringens*, and *E. coli* serotype O157:H7 for their potential in this area.^13^, ^14^, ^16^, ^63^, ^64^, ^76^, ^91^, ^93^, ^94^, ^99^

In addition to strategies aimed at editing the tumor ECM/glycocalyx using the enzymes mentioned in the previous sections, evidence also shows direct effects of enzymes on tumor cells. To this end, holistic screening approaches should focus on identifying multifaceted enzymes with extracellular editing activities and direct effects on cancer cells, as well as other potential mechanisms of action. Reports describe the antitumor activity of certain metalloproteases, such as serralysin and arazyme.^120^, ^121^ Arazyme, a metalloproteinase secreted by *Serratia proteamaculans*, showed dose‐dependent cytostatic activity. In addition, arazyme disrupted cell adhesion and potentially cell‐ECM signaling by degrading CD44. Notably, arazyme revealed antitumor activity in an animal model.^121^, ^122^ In addition to inhibiting cell proliferation, arazyme may have a direct cytotoxic effect.^123^, ^124^, ^125^ Furthermore, the literature has reported potential antitumor effects of other enzymes.^120^, ^126^ Although such enzymes may offer the advantage of acting through multiple mechanisms, these activities may arise from broad substrate cleavage rather than tumor‐selective mechanisms, potentially increasing the risks of off‐target activity. Comparative studies are required to determine whether direct antitumor effects can be achieved within the dose range required for extracellular editing and whether these doses preferentially affect cancer cells over normal cells. Therefore, a cautious interpretation is warranted, and further studies with the above‐mentioned research questions are needed to shed light on their potential use as multifaceted enzymes in combination with immunotherapeutics.

Many microorganisms utilize enzymes throughout their life cycles, whether for pathogenesis or physiological functions. A case in point is StcE, which was previously discussed and was further engineered to achieve the desired responses.^13^, ^100^ However, in nature, StcE facilitates the attachment of *E. coli* serotype O157:H7 to host cells, one of the most well‐recognized *E. coli* pathotypes responsible for global outbreaks of bloody diarrhea.^127^ A similar approach can also be observed in other studies.^14^, ^91^ These examples suggest that enzymes with established roles in microbial life cycles may provide useful starting points for extracellular‐editing strategies.^128^ In line with this idea, it is worth mentioning the important role of human microbiota in ECM remodeling; thus, bacterial enzymes known to participate in this process could be screened for their potential use in therapeutic ECM remodeling.^129^ Similarly, oral dysbiosis can lead to significant ECM remodeling, largely driven by a plethora of secreted enzymes.^130^, ^131^

Furthermore, chondroitin sulfate‐degrading enzymes represent another potentially useful but underexplored group. Among these, chondroitinase ABC (ChABC), a lyase derived from *Proteus vulgaris* that cleaves the chondroitin sulfate (CS) chains of proteoglycan molecules, has emerged as a promising therapeutic strategy in preclinical models of spinal cord injury.^132^, ^133^ ChABC also exhibits some degree of HA‐degrading activity, further supporting its potential for ECM modulation.^134^ The modification of chondroitin sulfate proteoglycans (CSPGs) by ChABC may represent a novel approach to prime the TME and to improve therapeutic outcomes, as CSPGs can also hinder therapeutic access.^135^, ^136^, ^137^ Similar to other ECM components, CSPGs, such as versican, neurocan, and CSPG4, accumulate in the tumor, supporting tumor growth and invasion by activating oncogenic pathways and shaping the tumor microenvironment.^138^, ^139^, ^140^, ^141^ The distinct CS composition of versican can either enable or block T cell movement; enzymatically editing these CS chains (e.g., with ChABC) may overcome inhibitory matrices and enhance immunotherapy potential.^135^ Notably, a more targeted approach using chondroitin C lyase has been proposed to selectively remove CS isomers and enhance immune infiltration, highlighting the importance of protein engineering and advanced screening platforms in remodeling the ECM.^135^, ^142^ Taken together, these examples suggest prioritizing new enzyme candidates based on a clear chain of evidence linking cleavage of a tumor‐relevant extracellular substrate to improved immune‐cell access, function, or response to immunotherapy. Enzymes supported only by biochemical plausibility outside this context should therefore remain classified as exploratory.

Computational methods may complement these screening efforts by identifying distant sequence or structural homologs and supporting predictions of enzyme function and substrate compatibility.^143^, ^144^, ^145^ They may also prioritize mutations that improve catalytic activity, substrate specificity, stability, solubility, and performance under tumor microenvironmental conditions. Generative protein‐design approaches may further propose amino acid sequences for existing or newly generated backbones that meet specified structural or catalytic constraints.^143^, ^146^ For example, one study combined artificial intelligence (AI)‐guided sequence redesign with directed evolution to improve botulinum neurotoxin protease stability, catalytic efficiency, and selectivity for a non‐native substrate while reducing activity toward the native substrate.^146^ However, applying these approaches to complex ECM and glycocalyx substrates will require experimental validation of their activity, selectivity, stability, and safety.

### 8.4 Immunogenicity of microbial enzymes

Identifying promising microbial enzymes for extracellular editing is an early step toward therapeutic development. Their foreign origin remains an important translational concern, as several approved enzymes of non‐human origin have been associated with hypersensitivity reactions and ADA formation in clinical practice.^79^, ^128^, ^147^, ^148^ Enzymes have been used as cytotoxic components of immunotoxins, as tools for local prodrug activation in antibody‐directed enzyme prodrug therapy, and, more recently, as modifiers of tumor‐cell surfaces and the tumor microenvironment.^149^, ^150^ These platforms apply antibody‐guided targeting principles similar to those used in antibody–drug conjugates (ADCs) to concentrate enzymatic activity at tumor‐associated sites and limit off‐target effects, but targeted delivery does not eliminate the intrinsic antigenicity of a foreign protein.^128^, ^151^ Enzyme‐based immunotoxins act primarily through cellular internalization, which may limit the duration of extracellular exposure.^149^ In contrast, enzymes designed to edit the ECM or glycocalyx must remain active in the extracellular space and may therefore experience more prolonged exposure to circulating antibodies and immune cells.^128^, ^152^ This distinct pharmacological context could increase the risk of adverse immune responses, particularly with repeated dosing. Experience with targeted enzyme therapeutics in oncology has shown that these responses are not limited to overt clinical reactions; enzyme‐specific antibodies may also accelerate clearance, neutralize catalytic activity, and thereby compromise both pharmacokinetics and pharmacodynamics.^152^, ^153^ Several strategies have been explored to mitigate enzyme immunogenicity, including PEGylation, transient immunosuppression with immunomodulatory agents, and protein engineering to remove or modify immunogenic epitopes. PEGylated forms of hyaluronidase and asparaginase provide relevant examples; however, this modification has not fully addressed the issue.^147^ Transient immunosuppression may reduce undesired reactions but introduces additional safety concerns, as immunosuppressants can increase the risk of serious adverse effects.^154^, ^155^ Immunogenicity may also be reduced by identifying and modifying the epitopes recognized by T‐ or B‐cells. However, such engineering must preserve protein folding, catalytic activity, stability, and substrate specificity. Most reported deimmunization efforts have focused on immunotoxins and other established enzymes with therapeutic use.^156^, ^157^ While these studies primarily relied on experimental epitope mapping and targeted mutagenesis, computational epitope‐prediction tools may offer new opportunities to identify and prioritize modifications that reduce therapeutic‐enzyme immunogenicity by limiting recognition by existing ADAs and the induction of new ADAs.^158^ In the literature reviewed here, we did not identify studies applying comparable strategies specifically to ECM‐ or glycocalyx‐editing enzymes, highlighting an important translational research gap.

### 8.5 Pharmaceutical development and regulatory considerations

Knowledge translation for public use requires extra considerations beyond discovery and clinical efficacy. From a pharmaceutical development standpoint, successful translation of extracellular editing modalities will require precise and sensitive quality control assays (QC), scalable manufacturing processes, and regulatory frameworks. Although several therapeutic enzymes are already covered by monographs in pharmacopeias (e.g., Alteplase),^159^ critical quality attributes (CQAs) and analytical strategies remain to be defined for most of the path cleansers discussed here. Existing frameworks may provide useful hints related to identity, purity, protein content, potency, process‐related impurities, aggregation, and stability. For the enzyme itself, potency should be evaluated using a validated assay representative of its intended biological activity and, where appropriate, expressed as specific activity relative to protein content. The choice of assay should remain product‐specific and be supported by mechanistic and development data. Comprehensive substrate‐selectivity, off‐target activity, serum stability, and immunogenicity assessments are primarily investigated at the product development level and are not usually considered in routine lot release. Beyond these enzyme‐specific considerations, incorporation into targeted conjugates or engineered cellular platforms may introduce additional quality attributes.

For antibody‐enzyme conjugates, experience with ADCs can provide a useful framework for defining additional quality attributes. In such therapeutics, conjugation should preserve both targeting and catalytic functions while maintaining conjugate integrity, since premature deconjugation may reduce localized activity and simultaneously increase exposure to catalytically active free enzyme. Furthermore, the enzyme‐to‐antibody ratio and levels of free enzyme or antibody may affect therapeutic activity and off‐target exposure and should therefore be appropriately checked, particularly for chemically conjugated products. As with other biotherapeutics, aggregation may also occur during manufacturing or storage. This phenomenon is important because aggregation may diminish enzyme activity while increasing the risk of immunogenicity and undesired immune reactions.^160^

Engineered cellular platforms that constitute enzymes require additional considerations, as enzymatic function becomes part of an already complex cellular product. Conventional cellular CQAs should be complemented by assessment of enzyme secretion or surface activity, as these functions may influence both product potency and safety.^161^, ^162^ For cells designed to secrete the enzyme, consistent production and catalytic activity should be demonstrated without compromising the underlying cellular potency. For surface‐displayed enzymes, the amount of enzyme carried by the cells, its retention on the cell surface, and cell‐associated catalytic activity may also be relevant, since insufficient loading or loss of anchoring could reduce localized remodeling. Separate introduction of the enzyme and immune‐cell receptor may also require consideration of consistency in co‐expression, as heterogeneous cell populations can arise.^163^, ^164^ These added functions also make batch‐to‐batch consistency particularly important, and controlling key process parameters (CPPs) is likely necessary to maintain consistent enzyme expression or secretion and overall CQA performance across lots. In conclusion, not all R&D activities would necessarily form part of routine lot release, as some may be better controlled through the manufacturing process or assessed during stability and product development studies.^164^

## AUTHOR CONTRIBUTIONS

**NM** developed the main conceptual framework. **NM** and **MA** contributed to writing the original draft and to reviewing and editing the manuscript. **NM** designed the graphical illustrations and prepared the tables.

## CONFLICT OF INTEREST STATEMENT

The authors report there are no competing interests to declare.

## Data Availability

Data sharing not applicable to this article as no data sets were generated or analyzed during the current study.

## References

[1] Karamanos NK , Theocharis AD , Piperigkou Z , et al. A guide to the composition and functions of the extracellular matrix. FEBS J. 2021;288:6850‐6912. 10.1111/febs.15776 33605520

[2] Cox TR . The matrix in cancer. Nat Rev Cancer. 2021;21:217‐238. 10.1038/s41568-020-00329-7 33589810

[3] Henke E , Nandigama R , Ergün S . Extracellular matrix in the tumor microenvironment and its impact on cancer therapy. Front Mol Biosci. 2020;6:160. 10.3389/fmolb.2019.00160 PMC7025524 32118030

[4] Chen Z , Han F , Du Y , Shi H , Zhou W . Hypoxic microenvironment in cancer: molecular mechanisms and therapeutic interventions. Signal Transduct Target Ther. 2023;8:70. 10.1038/s41392-023-01332-8 PMC9935926 36797231

[5] Cho Y , Doh J . The extracellular matrix in solid tumor immunotherapy. Trends Immunol. 2024;45:705‐714. 10.1016/j.it.2024.07.009 39164157

[6] He X , Lee B , Jiang Y . Extracellular matrix in cancer progression and therapy. Med Rev. 2022;2:125‐139. 10.1515/mr-2021-0028 PMC10471113 37724245

[7] Flies DB , Langermann S , Jensen C , Karsdal MA , Willumsen N . Regulation of tumor immunity and immunotherapy by the tumor collagen extracellular matrix. Front Immunol. 2023;14:1199513. 10.3389/fimmu.2023.1199513 PMC10470046 37662958

[8] Ghasempour S , Freeman SA . The glycocalyx and immune evasion in cancer. FEBS J. 2023;290:55‐65. 10.1111/febs.16236 34665926

[9] Huang J , Zhang L , Wan D , et al. Extracellular matrix and its therapeutic potential for cancer treatment. Signal Transduct Target Ther. 2021;6:153. 10.1038/s41392-021-00544-0 PMC8062524 33888679

[10] Vlodavsky I , Friedmann Y , Elkin M , et al. Mammalian heparanase: gene cloning, expression and function in tumor progression and metastasis. Nat Med. 1999;5:793‐802. 10.1038/10518 10395325

[11] Eikenes L , Bruland ØS , Brekken C , Davies CL . Collagenase increases the transcapillary pressure gradient and improves the uptake and distribution of monoclonal antibodies in human osteosarcoma xenografts. Cancer Res. 2004;64:4768‐4773. 10.1158/0008-5472.CAN-03-1472 15256445

[12] Burkhard T , Milne E , Qian K , Campagnolo P , Santamaria S . Proteolytic remodelling of the extracellular matrix by pericytes. FEBS J. 2026;293:3899‐3953. 10.1111/febs.70569 PMC13326524 42095275

[13] Pedram K , Shon DJ , Tender GS , et al. Design of a mucin‐selective protease for targeted degradation of cancer‐associated mucins. Nat Biotechnol. 2024;42:597‐607. 10.1038/s41587-023-01840-6 PMC11018308 37537499

[14] Gray MA , Stanczak MA , Mantuano NR , et al. Targeted glycan degradation potentiates the anticancer immune response in vivo. Nat Chem Biol. 2020;16:1376‐1384. 10.1038/s41589-020-0622-x PMC7727925 32807964

[15] Prakash J , Shaked Y . The interplay between extracellular matrix remodeling and cancer therapeutics. Cancer Discov. 2024;14:1375‐1388. 10.1158/2159-8290.CD-24-0002 PMC11294818 39091205

[16] Yang Z , Hou Y , Grande G , et al. Targeted desialylation and cytolysis of tumour cells by fusing a sialidase to a bispecific T‐cell engager. Nat Biomed Eng. 2024;8:499‐512. 10.1038/s41551-024-01202-w PMC11577304 38693431

[17] Yuan Z , Li Y , Zhang S , et al. Extracellular matrix remodeling in tumor progression and immune escape: from mechanisms to treatments. Mol Cancer. 2023;22:48. 10.1186/s12943-023-01744-8 PMC10007858 36906534

[18] Ryan AT , Kim M , Lim K . Immune cell migration to cancer. Cells. 2024;13:844. 10.3390/cells13100844 PMC11120175 38786066

[19] Jayatilleke KM , Hulett MD . Heparanase and the hallmarks of cancer. J Transl Med. 2020;18:453. 10.1186/s12967-020-02624-1 PMC7706218 33256730

[20] Nagarajan A , Malvi P , Wajapeyee N . Heparan sulfate and heparan sulfate proteoglycans in cancer initiation and progression. Front Endocrinol. 2018;9:483. 10.3389/fendo.2018.00483 PMC6118229 30197623

[21] Naparstek Y , Cohen IR , Fuks Z , Vlodavsky I . Activated T lymphocytes produce a matrix‐degrading heparan sulphate endoglycosidase. Nature. 1984;310:241‐244. 10.1038/310241a0 6205275

[22] Mayfosh AJ , Baschuk N , Hulett MD . Leukocyte heparanase: a double‐edged sword in tumor progression. Front Oncol. 2019;9:331. 10.3389/fonc.2019.00331 PMC6501466 31110966

[23] Caruana I , Savoldo B , Hoyos V , et al. Heparanase promotes tumor infiltration and antitumor activity of CAR‐redirected T lymphocytes. Nat Med. 2015;21:524‐529. 10.1038/nm.3833 PMC4425589 25849134

[24] Liborio‐Ramos S , Quiros‐Fernandez I , Ilan N , et al. An integral membrane constitutively active heparanase enhances the tumor infiltration capability of NK cells. Onco Targets Ther. 2025;14:2437917. 10.1080/2162402X.2024.2437917 PMC11633225 39651893

[25] Coombe DR , Gandhi NS . Heparanase: a challenging cancer drug target. Front Oncol. 2019;9:1316. 10.3389/fonc.2019.01316 PMC6892829 31850210

[26] Mitra A , Barua A , Huang L , Ganguly S , Feng Q , He B . From bench to bedside: the history and progress of CAR T cell therapy. Front Immunol. 2023;14:1188049. 10.3389/fimmu.2023.1188049 PMC10225594 37256141

[27] Dias J , Garcia J , Agliardi G , Roddie C . CAR‐T cell manufacturing landscape—lessons from the past decade and considerations for early clinical development. Mol Ther Methods Clin Dev. 2024;32:101250. 10.1016/j.omtm.2024.101250 PMC11088187 38737799

[28] Gingis‐Velitski S , Zetser A , Flugelman MY , Vlodavsky I , Ilan N . Heparanase induces endothelial cell migration via protein kinase B/Akt activation. J Biol Chem. 2004;279:23536‐23541. 10.1074/jbc.M400554200 15044433

[29] Vlodavsky I , Singh P , Boyango I , et al. Heparanase: from basic research to therapeutic applications in cancer and inflammation. Drug Resist Updat. 2016;29:54‐75. 10.1016/j.drup.2016.10.001 PMC5447241 27912844

[30] Lu L , Xie M , Yang B , Zhao W‐b , Cao J . Enhancing the safety of CAR‐T cell therapy: synthetic genetic switch for spatiotemporal control. Sci Adv. 2024;10:eadj6251. 10.1126/sciadv.adj6251 PMC10889354 38394207

[31] Liu M , Tolg C , Turley E . Dissecting the dual nature of Hyaluronan in the tumor microenvironment. Front Immunol. 2019;10:947. 10.3389/fimmu.2019.00947 PMC6522846 31134064

[32] Spinelli FM , Vitale DL , Sevic I , Alaniz L . Hyaluronan in the tumor microenvironment. Adv Exp Med Biol. 2020;1245:67‐83. 10.1007/978-3-030-40146-7_3 32266653

[33] Thompson CB , Shepard HM , O'Connor PM , et al. Enzymatic depletion of tumor hyaluronan induces antitumor responses in preclinical animal models. Mol Cancer Ther. 2010;9:3052‐3064. 10.1158/1535-7163.MCT-10-0470 20978165

[34] Jacobetz MA , Chan DS , Neesse A , et al. Hyaluronan impairs vascular function and drug delivery in a mouse model of pancreatic cancer. Gut. 2013;62:112‐120. 10.1136/gutjnl-2012-302529 PMC3551211 22466618

[35] Liu Z , Hou P , Fang J , et al. Mesenchymal stromal cells confer breast cancer doxorubicin resistance by producing hyaluronan. Oncogene. 2023;42:3221‐3235. 10.1038/s41388-023-02837-w 37704784

[36] Singha NC , Nekoroski T , Zhao C , et al. Tumor‐associated hyaluronan limits efficacy of monoclonal antibody therapy. Mol Cancer Ther. 2015;14:523‐532. 10.1158/1535-7163.MCT-14-0580 25512619

[37] Provenzano PP , Cuevas C , Chang AE , Goel VK , Von Hoff DD , Hingorani SR . Enzymatic targeting of the stroma ablates physical barriers to treatment of pancreatic ductal adenocarcinoma. Cancer Cell. 2012;21:418‐429. 10.1016/j.ccr.2012.01.007 PMC3371414 22439937

[38] Ramanathan RK , McDonough SL , Philip PA , et al. Phase IB/II randomized study of FOLFIRINOX plus pegylated recombinant human hyaluronidase versus FOLFIRINOX alone in patients with metastatic pancreatic adenocarcinoma: SWOG S1313. J Clin Oncol. 2019;37:1062‐1069. 10.1200/JCO.18.01295 PMC6494359 30817250

[39] Van Cutsem E , Tempero MA , Sigal D , et al. Randomized phase III trial of pegvorhyaluronidase alfa with nab‐paclitaxel plus gemcitabine for patients with hyaluronan‐high metastatic pancreatic adenocarcinoma. J Clin Oncol. 2020;38:3185‐3194. 10.1200/JCO.20.00590 PMC7499614 32706635

[40] McAtee CO , Barycki JJ , Simpson MA . Emerging roles for hyaluronidase in cancer metastasis and therapy. Adv Cancer Res. 2014;123:1‐34. 10.1016/B978-0-12-800092-2.00001-0 PMC4445717 25081524

[41] Kovar JL , Johnson MA , Volcheck WM , Chen J , Simpson MA . Hyaluronidase expression induces prostate tumor metastasis in an orthotopic mouse model. Am J Pathol. 2006;169:1415‐1426. 10.2353/ajpath.2006.060324 PMC1698854 17003496

[42] Kultti A , Li X , Jiang P , Thompson CB , Frost GI , Shepard HM . Therapeutic targeting of hyaluronan in the tumor stroma. Cancers. 2012;4:873‐903. 10.3390/cancers4030873 PMC3712709 24213471

[43] Zhao R , Cui Y , Zheng Y , et al. Human hyaluronidase PH20 potentiates the antitumor activities of mesothelin‐specific CAR‐T cells against gastric cancer. Front Immunol. 2021;12:660488. 10.3389/fimmu.2021.660488 PMC8313856 34326835

[44] Xiong X , Xi J , Liu Q , et al. Co‐expression of IL‐7 and PH20 promote anti‐GPC3 CAR‐T tumour suppressor activity in vivo and in vitro. Liver Int. 2021;41:1033‐1043. 10.1111/liv.14771 33347692

[45] Zhao Y , Dong Y , Yang S , et al. Bioorthogonal equipping CAR‐T cells with hyaluronidase and checkpoint blocking antibody for enhanced solid tumor immunotherapy. ACS Cent Sci. 2022;8:603‐614. 10.1021/acscentsci.2c00163 PMC9136969 35647274

[46] Hong Y , Nam G‐H , Koh E , et al. Exosome as a vehicle for delivery of membrane protein therapeutics, PH20, for enhanced tumor penetration and antitumor efficacy. Adv Funct Mater. 2018;28:1703074.

[47] Hong Y , Kim YK , Kim GB , et al. Degradation of tumour stromal hyaluronan by small extracellular vesicle‐PH20 stimulates CD103(+) dendritic cells and in combination with PD‐L1 blockade boosts anti‐tumour immunity. J Extracell Vesicles. 2019;8:1670893. 10.1080/20013078.2019.1670893 PMC6781230 31632619

[48] Xiao Z , Tan Y , Cai Y , et al. Nanodrug removes physical barrier to promote T‐cell infiltration for enhanced cancer immunotherapy. J Control Release. 2023;356:360‐372. 10.1016/j.jconrel.2023.02.029 36871644

[49] Guan X , Chen J , Hu Y , et al. Highly enhanced cancer immunotherapy by combining nanovaccine with hyaluronidase. Biomaterials. 2018;171:198‐206. 10.1016/j.biomaterials.2018.04.039 29698869

[50] Ott PA , Wu CJ . Cancer vaccines: steering T cells down the right path to eradicate tumors. Cancer Discov. 2019;9:476‐481. 10.1158/2159-8290.CD-18-1357 PMC7067230 30862723

[51] Liu J , Fu M , Wang M , Wan D , Wei Y , Wei X . Cancer vaccines as promising immuno‐therapeutics: platforms and current progress. J Hematol Oncol. 2022;15:28. 10.1186/s13045-022-01247-x PMC8931585 35303904

[52] Rømer AMA , Thorseth ML , Madsen DH . Immune modulatory properties of collagen in cancer. Front Immunol. 2021;12:791453. 10.3389/fimmu.2021.791453 PMC8692250 34956223

[53] Frantz C , Stewart KM , Weaver VM . The extracellular matrix at a glance. J Cell Sci. 2010;123:4195‐4200. 10.1242/jcs.023820 PMC2995612 21123617

[54] Salmon H , Franciszkiewicz K , Damotte D , et al. Matrix architecture defines the preferential localization and migration of T cells into the stroma of human lung tumors. J Clin Invest. 2012;122:899‐910. 10.1172/JCI45817 PMC3287213 22293174

[55] Lebbink RJ , de Ruiter T , Adelmeijer J , et al. Collagens are functional, high affinity ligands for the inhibitory immune receptor LAIR‐1. J Exp Med. 2006;203:1419‐1425. 10.1084/jem.20052554 PMC2118306 16754721

[56] Peng DH , Rodriguez BL , Diao L , et al. Collagen promotes anti‐PD‐1/PD‐L1 resistance in cancer through LAIR1‐dependent CD8(+) T cell exhaustion. Nat Commun. 2020;11:4520. 10.1038/s41467-020-18298-8 PMC7481212 32908154

[57] Zhang X , Zhao Y , Chen X . Collagen extracellular matrix promotes gastric cancer immune evasion by activating IL4I1‐AHR signaling. Transl Oncol. 2024;49:102113. 10.1016/j.tranon.2024.102113 PMC11402615 39216468

[58] Jain RK . Delivery of molecular medicine to solid tumors: lessons from in vivo imaging of gene expression and function. J Control Release. 2001;74:7‐25. 10.1016/s0168-3659(01)00306-6 11489479

[59] Li Z , Mo F , Guo K , et al. Nanodrug‐bacteria conjugates‐mediated oncogenic collagen depletion enhances immune checkpoint blockade therapy against pancreatic cancer. Med. 2024;5:348‐367.e347. 10.1016/j.medj.2024.02.012 38521069

[60] Chen Y , Yang S , Tavormina J , et al. Oncogenic collagen I homotrimers from cancer cells bind to α3β1 integrin and impact tumor microbiome and immunity to promote pancreatic cancer. Cancer Cell. 2022;40:818‐834.e819. 10.1016/j.ccell.2022.06.011 PMC9831277 35868307

[61] Kalia P , Thakur P , Kumar R . Advancing precision therapeutics integrating microbiota biomarkers and nanotechnology for targeted drug delivery: insights from case studies. In: Singh SK , Vamanu E , Rai SN , Singh AK , eds. Microbiota Profiling for Precision Medicine: Biomarker‐Targeted Drug Delivery Strategies. Springer Nature Singapore; 2025:239‐280.

[62] Weldon JE , Pastan I . A guide to taming a toxin–recombinant immunotoxins constructed from pseudomonas exotoxin a for the treatment of cancer. FEBS J. 2011;278:4683‐4700. 10.1111/j.1742-4658.2011.08182.x PMC3179548 21585657

[63] Li H‐R , Ye B‐C . Engineered probiotic‐mediated intratumoral delivery and controlled release of bacterial collagenase for cancer therapy. Synth Syst Biotechnol. 2025;10:226‐236. 10.1016/j.synbio.2024.09.001 PMC11585803 39582690

[64] Pan A , Wang Z , Chen B , et al. Localized co‐delivery of collagenase and trastuzumab by thermosensitive hydrogels for enhanced antitumor efficacy in human breast xenograft. Drug Deliv. 2018;25:1495‐1503. 10.1080/10717544.2018.1474971 PMC6058501 29943651

[65] Zhao Z , Li Q , Qu C , et al. A collagenase nanogel backpack improves CAR‐T cell therapy outcomes in pancreatic cancer. Nat Nanotechnol. 2025;20:1131‐1141. 10.1038/s41565-025-01924-1 40389641

[66] Hartmann N , Giese NA , Giese T , et al. Prevailing role of contact guidance in intrastromal T‐cell trapping in human pancreatic cancer. Clin Cancer Res. 2014;20:3422‐3433. 10.1158/1078-0432.CCR-13-2972 24763614

[67] Zhang T , Ren Y , Yang P , Wang J , Zhou H . Cancer‐associated fibroblasts in pancreatic ductal adenocarcinoma. Cell Death Dis. 2022;13:897. 10.1038/s41419-022-05351-1 PMC9596464 36284087

[68] Feig C , Jones JO , Kraman M , et al. Targeting CXCL12 from FAP‐expressing carcinoma‐associated fibroblasts synergizes with anti‐PD‐L1 immunotherapy in pancreatic cancer. Proc Natl Acad Sci U S A. 2013;110:20212‐20217. 10.1073/pnas.1320318110 PMC3864274 24277834

[69] Ene‐Obong A , Clear AJ , Watt J , et al. Activated pancreatic stellate cells sequester CD8+ T cells to reduce their infiltration of the juxtatumoral compartment of pancreatic ductal adenocarcinoma. Gastroenterology. 2013;145:1121‐1132. 10.1053/j.gastro.2013.07.025 PMC3896919 23891972

[70] Saur D , Seidler B , Schneider G , et al. CXCR4 expression increases liver and lung metastasis in a mouse model of pancreatic cancer. Gastroenterology. 2005;129:1237‐1250. 10.1053/j.gastro.2005.06.056 16230077

[71] Domanska UM , Kruizinga RC , Nagengast WB , et al. A review on CXCR4/CXCL12 axis in oncology: no place to hide. Eur J Cancer. 2013;49:219‐230. 10.1016/j.ejca.2012.05.005 22683307

[72] Xu J , Chu T , Liu Z , et al. Cancer‐associated fibroblast programs across cancers: functional axes and double‐edged roles in pancreatic ductal adenocarcinoma. Genes Dis. 2026;102275. 10.1016/j.gendis.2026.102275.

[73] Chen Y , Kim J , Yang S , et al. Type I collagen deletion in αSMA(+) myofibroblasts augments immune suppression and accelerates progression of pancreatic cancer. Cancer Cell. 2021;39:548‐565.e546. 10.1016/j.ccell.2021.02.007 PMC8423173 33667385

[74] Rhim AD , Oberstein PE , Thomas DH , et al. Stromal elements act to restrain, rather than support, pancreatic ductal adenocarcinoma. Cancer Cell. 2014;25:735‐747. 10.1016/j.ccr.2014.04.021 PMC4096698 24856585

[75] Spada S , Tocci A , Di Modugno F , Nisticò P . Fibronectin as a multiregulatory molecule crucial in tumor matrisome: from structural and functional features to clinical practice in oncology. J Exp Clin Cancer Res. 2021;40:102. 10.1186/s13046-021-01908-8 PMC7972229 33731188

[76] Zhang Y , Pei P , Zhou H , et al. Nattokinase‐mediated regulation of tumor physical microenvironment to enhance chemotherapy, radiotherapy, and CAR‐T therapy of solid tumor. ACS Nano. 2023;17:7475‐7486. 10.1021/acsnano.2c12463 37057972

[77] Urano T , Ihara H , Umemura K , et al. The profibrinolytic enzyme subtilisin NAT purified from Bacillus subtilis cleaves and inactivates plasminogen activator inhibitor type 1. J Biol Chem. 2001;276:24690‐24696. 10.1074/jbc.M101751200 11325965

[78] Chen H , McGowan EM , Ren N , et al. Nattokinase: a promising alternative in prevention and treatment of cardiovascular diseases. Biomark Insights. 2018;13:1177271918785130. 10.1177/1177271918785130 PMC6043915 30013308

[79] Suzuki K , Nakamura M , Sato N , Futamura K , Matsunaga K , Yagami A . Nattokinase (Bac s 1), a subtilisin family serine protease, is a novel allergen contained in the traditional Japanese fermented food natto. Allergol Int. 2023;72:279‐285. 10.1016/j.alit.2022.11.010 36517353

[80] Büll C , Stoel MA , den Brok MH , Adema GJ . Sialic acids sweeten a tumor's life. Cancer Res. 2014;74:3199‐3204. 10.1158/0008-5472.CAN-14-0728 24830719

[81] Cohen M , Elkabets M , Perlmutter M , et al. Sialylation of 3‐methylcholanthrene‐induced fibrosarcoma determines antitumor immune responses during immunoediting. J Immunol. 2010;185:5869‐5878. 10.4049/jimmunol.1001635 20956342

[82] Zhou X , Chi K , Zhang C , Liu Q , Yang G . Sialylation: a cloak for tumors to trick the immune system in the microenvironment. Biology. 2023;12:832. 10.3390/biology12060832 PMC10294807 37372117

[83] Macauley MS , Crocker PR , Paulson JC . Siglec‐mediated regulation of immune cell function in disease. Nat Rev Immunol. 2014;14:653‐666. 10.1038/nri3737 PMC4191907 25234143

[84] Hudak JE , Canham SM , Bertozzi CR . Glycocalyx engineering reveals a Siglec‐based mechanism for NK cell immunoevasion. Nat Chem Biol. 2014;10:69‐75. 10.1038/nchembio.1388 PMC3893890 24292068

[85] Christie DR , Shaikh FM , Lucas JA , Lucas JA 3rd , Bellis SL . ST6Gal‐I expression in ovarian cancer cells promotes an invasive phenotype by altering integrin glycosylation and function. J Ovarian Res. 2008;1:3. 10.1186/1757-2215-1-3 PMC2584051 19014651

[86] Ou L , He X , Liu N , et al. Sialylation of FGFR1 by ST6Gal‐I overexpression contributes to ovarian cancer cell migration and chemoresistance. Mol Med Rep. 2020;21:1449‐1460. 10.3892/mmr.2020.10951 PMC7003046 32016470

[87] Pearce OM , Läubli H . Sialic acids in cancer biology and immunity. Glycobiology. 2016;26:111‐128. 10.1093/glycob/cwv097 26518624

[88] Sharma MR , Johnson ML , Puzanov I , et al. 758 GLIMMER‐01: phase 1/2 trial of a first‐in‐class bi‐sialidase (E‐602) in combination with cemiplimab in patients with PD‐(L)1‐resistant solid tumors. J Immunother Cancer. 2024;12:A862.

[89] Garabedian BM , Bashian EE , Wang X , Thompson AJ , Paulson JC . Targeting sialidase to PD1 enhances T cell function and tumor control. ACS Cent Sci. 2025;11:1417‐1427. 10.1021/acscentsci.5c00510 PMC12395300 40893963

[90] Mensah SA , Harding IC , Zhang M , et al. Metastatic cancer cell attachment to endothelium is promoted by endothelial glycocalyx sialic acid degradation. AIChE J. 2019;65:e16634. 10.1002/aic.16634 PMC6668365 31367063

[91] Xiao H , Woods EC , Vukojicic P , Bertozzi CR . Precision glycocalyx editing as a strategy for cancer immunotherapy. Proc Natl Acad Sci U S A. 2016;113:10304‐10309. 10.1073/pnas.1608069113 PMC5027407 27551071

[92] Peng Z , Hodgson K , Fisher M , et al. Targeting Siglec‐engaging immunosuppressive sialoglycans to suppress prostate cancer bone metastasis. Br J Cancer. 2026;135:1164‐1177. 10.1038/s41416-026-03544-5 PMC13578327 42443559

[93] Stanczak MA , Rodrigues Mantuano N , Kirchhammer N , et al. Targeting cancer glycosylation repolarizes tumor‐associated macrophages allowing effective immune checkpoint blockade. Sci Transl Med. 2022;14:eabj1270. 10.1126/scitranslmed.abj1270 PMC9812757 36322632

[94] Tong Y , Chen R , Lu X , et al. A nanobody‐enzyme fusion protein targeting PD‐L1 and sialic acid exerts anti‐tumor effects by C‐type lectin pathway‐mediated tumor associated macrophages repolarizing. Int J Biol Macromol. 2025;298:139953. 10.1016/j.ijbiomac.2025.139953 39824395

[95] Jafari M , Maali A , Shokrgozar MA , Sharifzadeh Z . Editorial: Nanobody‐based cancer immunotherapy and immunoimaging. Front Immunol. 2023;14:1213386. 10.3389/fimmu.2023.1213386 PMC10227555 37261357

[96] Edgar LJ , Thompson AJ , Vartabedian VF . Sialic acid ligands of CD28 suppress costimulation of T cells. ACS Cent Sci. 2021;7:1508‐1515. 10.1021/acscentsci.1c00525 PMC8461770 34584952

[97] Goebeler M‐E , Bargou RC . T cell‐engaging therapies—BiTEs and beyond. Nat Rev Clin Oncol. 2020;17:418‐434. 10.1038/s41571-020-0347-5 32242094

[98] Szijj PA , Gray MA , Ribi MK , et al. Chemical generation of checkpoint inhibitory T cell engagers for the treatment of cancer. Nat Chem. 2023;15:1636‐1647. 10.1038/s41557-023-01280-4 PMC10624612 37488375

[99] Durgin JS , Thokala R , Johnson L , et al. Enhancing CAR T function with the engineered secretion of *C. perfringens* neuraminidase. Mol Ther. 2022;30:1201‐1214. 10.1016/j.ymthe.2021.11.014 PMC8899523 34813961

[100] Park S , Colville MJ , Paek JH , et al. Immunoengineering can overcome the glycocalyx armour of cancer cells. Nat Mater. 2024;23:429‐438. 10.1038/s41563-024-01808-0 PMC11471287 38361041

[101] Wu J , Wang X , Huang Y , et al. Targeted glycan degradation potentiates cellular immunotherapy for solid tumors. Proc Natl Acad Sci U S A. 2023;120:e2300366120. 10.1073/pnas.2300366120 PMC10515149 37695897

[102] Barkal AA , Brewer RE , Markovic M , et al. CD24 signalling through macrophage Siglec‐10 is a target for cancer immunotherapy. Nature. 2019;572:392‐396. 10.1038/s41586-019-1456-0 PMC6697206 31367043

[103] Liu Y , Wang Y , Yang Y , et al. Emerging phagocytosis checkpoints in cancer immunotherapy. Signal Transduct Target Ther. 2023;8:104. 10.1038/s41392-023-01365-z PMC9990587 36882399

[104] Gatlin W , Chang J‐K , Cao L , et al. Abstract 7158: human sialidase‐armed anti‐B7‐H3 antibody that enhances innate and adaptive antitumor immune responses. Cancer Res. 2026;86:7158.

[105] Hollingsworth MA , Swanson BJ . Mucins in cancer: protection and control of the cell surface. Nat Rev Cancer. 2004;4:45‐60. 10.1038/nrc1251 14681689

[106] Kufe DW . Mucins in cancer: function, prognosis and therapy. Nat Rev Cancer. 2009;9:874‐885. 10.1038/nrc2761 PMC2951677 19935676

[107] Geng X , Liu S , Pan Y , Ge Y , Rao L . Mucinase‐engineered cell membrane nanovesicles degrade the glycocalyx shield to potentiate antitumor immunity. Proc Natl Acad Sci U S A. 2026;123:e2529792123. 10.1073/pnas.2529792123 PMC13213970 42133749

[108] Yousefi‐Hashemabad MJ , Kamroo A , Rezvanimehr A , et al. CD47/SIRPα immune checkpoint modulation: a synergistic strategy for next‐generation CAR therapies. Mol Cancer Ther. 2026;25:912‐923. 10.1158/1535-7163.MCT-25-0531 41517888

[109] Yu S , Wang S , Wang X , Xu X . The axis of tumor‐associated macrophages, extracellular matrix proteins, and cancer‐associated fibroblasts in oncogenesis. Cancer Cell Int. 2024;24:335. 10.1186/s12935-024-03518-8 PMC11459962 39375726

[110] Chauhan VP , Martin JD , Liu H , et al. Angiotensin inhibition enhances drug delivery and potentiates chemotherapy by decompressing tumour blood vessels. Nat Commun. 2013;4:2516. 10.1038/ncomms3516 PMC3806395 24084631

[111] Borst R , Meyaard L , Pascoal Ramos MI . Understanding the matrix: collagen modifications in tumors and their implications for immunotherapy. J Transl Med. 2024;22:382. 10.1186/s12967-024-05199-3 PMC11040975 38659022

[112] Jiang H , Liu X , Knolhoff BL , et al. Development of resistance to FAK inhibition in pancreatic cancer is linked to stromal depletion. Gut. 2020;69:122‐132. 10.1136/gutjnl-2018-317424 PMC7167297 31076405

[113] Mishra P , Khan A , Tungare K . Immunotherapy modeling: harnessing the immune system in chronic disease treatment. In: Rai SN , Singh SK , Singh V , eds. Advancements in Modeling‐Based Therapeutics and Technology for Chronic Diseases. Academic Press; 2026:253‐282.

[114] Rampal A , Peyton SR , Vachet RW . MALDI mass spectrometry imaging of extracellular matrix proteins. Analyst. 2025;150:3247‐3256. 10.1039/d5an00556f PMC12426872 40643895

[115] Padubidri SR , Das S , Sreelakshmi KV , Shetty KS , Dehury B . Personalized medicine and preventive health: digital twins and predictive analytics. In: Rai SN , Singh AK , Singh P , eds. Advancing Early Diagnosis in Chronic Diseases. Academic Press; 2026:153‐176.

[116] Campos‐Parra AD , Puente‐Rivera J , López‐Camarillo C , et al. The stiff side of cancer: how matrix mechanics rewrites non‐coding RNA expression programs. Non‐coding RNA. 2026;12:7. 10.3390/ncrna12010007 PMC12943065 41745140

[117] Nelluri KDD , Lakshmi K , Kommoju M , et al. Targeting the extracellular matrix in precision medicine: opportunities for diagnostics and therapeutics. Mol Biol Rep. 2026;53:479. 10.1007/s11033-026-11624-1 41801504

[118] Anas M . Cancer detection: screening tools and biomarker discoveries. In: Rai SN , Singh AK , Singh P , eds. Advancing Early Diagnosis in Chronic Diseases. Academic Press; 2026:55‐86.

[119] Prasanth T . Advancements in biomarker discovery and validation for targeted therapies. In: Rai SN , Singh SK , Singh V , eds. Advancements in Modeling‐Based Therapeutics and Technology for Chronic Diseases. Academic Press; 2026:135‐164.

[120] Araghi A , Hashemi S , Sepahi AA , Faramarzi MA , Amin M . Purification and study of anti‐cancer effects of *Serratia marcescens* serralysin. Iran J Microbiol. 2019;11:320‐327. PMC6829104 31719964

[121] Pereira FV , Ferreira‐Guimarães CA , Paschoalin T , et al. A natural bacterial‐derived product, the metalloprotease Arazyme, inhibits metastatic murine melanoma by inducing MMP‐8 cross‐reactive antibodies. PLoS One. 2014;9:e96141. 10.1371/journal.pone.0096141 PMC4005744 24788523

[122] Kwak J , Lee K , Shin DH , et al. Biochemical and genetic characterization of arazyme, an extracellular metalloprotease produced from *Serratia proteamaculans* HY‐3. J Microbiol Biotechnol. 2007;17:761‐768. 18051297

[123] Ghadaksaz A , Imani Fooladi AA , Mahmoodzadeh Hosseini H , Nejad Satari T , Amin M . ARA‐linker‐TGFαL3: a novel chimera protein to target breast cancer cells. Med Oncol. 2021;38:96. 10.1007/s12032-021-01546-2 34273028

[124] Rahmani F , Ajoudanifar H , Arbab Soleimani N , Imani Fooladi AA . Targeted therapies in HER2‐positive breast cancer with receptor‐redirected arazyme‐linker‐herceptin as a novel fusion protein. Breast Cancer. 2024;31:1101‐1113. 10.1007/s12282-024-01625-y 39122876

[125] Yahyaee Z , Shahpari M , Mousavi Ghahfarrokhi SS , et al. Cloning and expression of recombinant arazyme with anti‐inflammatory and anti‐breast cancer potential. Arch Microbiol. 2024;206:319. 10.1007/s00203-024-04051-y 38907853

[126] Mahapatra R , Paul T . The emergence of microbial protease as a potential anti‐cancer agent. EJMO. 2024;8:394‐401.

[127] Grys TE , Siegel MB , Lathem WW , Welch RA . The StcE protease contributes to intimate adherence of enterohemorrhagic *Escherichia coli* O157:H7 to host cells. Infect Immun. 2005;73:1295‐1303. 10.1128/IAI.73.3.1295-1303.2005 PMC1064933 15731026

[128] Tender GS , Bertozzi CR . Bringing enzymes to the proximity party. RSC Chem Biol. 2023;4:986‐1002. doi:10.1039/D3CB00084B PMC10685825 38033727

[129] Porras AM , Zhou H , Shi Q , Xiao X , Longman R , Brito IL . Inflammatory bowel disease‐associated gut commensals degrade components of the extracellular matrix. mBio. 2022;13:e02201‐02222. 10.1128/mbio.02201-22 PMC9765649 36445085

[130] Iwasaki M , Usui M , Ariyoshi W , et al. Evaluation of the ability of the trypsin‐like peptidase activity assay to detect severe periodontitis. PLoS One. 2021;16:e0256538. 10.1371/journal.pone.0256538 PMC8378714 34415974

[131] Fouillen A , Grenier D , Barbeau J , Baron C , Moffatt P , Nanci A . Selective bacterial degradation of the extracellular matrix attaching the gingiva to the tooth. Eur J Oral Sci. 2019;127:313‐322. 10.1111/eos.12623 PMC6771947 31230388

[132] Yamagata T , Saito H , Habuchi O , Suzuki S . Purification and properties of bacterial chondroitinases and chondrosulfatases. J Biol Chem. 1968;243:1523‐1535. 5647268

[133] Crespo D , Asher RA , Lin R , Rhodes KE , Fawcett JW . How does chondroitinase promote functional recovery in the damaged CNS? Exp Neurol. 2007;206:159‐171. 10.1016/j.expneurol.2007.05.001 17572406

[134] Galtrey CM , Asher RA , Nothias F , Fawcett JW . Promoting plasticity in the spinal cord with chondroitinase improves functional recovery after peripheral nerve repair. Brain. 2007;130:926‐939. 10.1093/brain/awl372 17255150

[135] Hirani P , McDermott J , Rajeeve V , et al. Versican associates with tumor immune phenotype and limits T‐cell trafficking via chondroitin sulfate. Cancer Res Commun. 2024;4:970‐985. 10.1158/2767-9764.CRC-23-0548 PMC10989462 38517140

[136] Kim Y , Lee HG , Dmitrieva N , Kim J , Kaur B , Friedman A . Choindroitinase ABC I‐mediated enhancement of oncolytic virus spread and antitumor efficacy: a mathematical model. PLoS One. 2014;9:e102499. 10.1371/journal.pone.0102499 PMC4105445 25047810

[137] Dmitrieva N , Yu L , Viapiano M , et al. Chondroitinase ABC I‐mediated enhancement of oncolytic virus spread and antitumor efficacy. Clin Cancer Res. 2011;17:1362‐1372. 10.1158/1078-0432.CCR-10-2213 PMC3140790 21177410

[138] Su Z , Kishida S , Tsubota S , et al. Neurocan, an extracellular chondroitin sulfate proteoglycan, stimulates neuroblastoma cells to promote malignant phenotypes. Oncotarget. 2017;8:106296‐106310. 10.18632/oncotarget.22435 PMC5739734 29290949

[139] Mellai M , Casalone C , Corona C , et al. Chondroitin sulphate proteoglycans in the tumour microenvironment. Adv Exp Med Biol. 2020;1272:73‐92. 10.1007/978-3-030-48457-6_5 32845503

[140] Du WW , Yang W , Yee AJ . Roles of versican in cancer biology – tumorigenesis, progression and metastasis. Histol Histopathol. 2013;28:701‐713. 10.14670/HH-28.701 23519970

[141] Pearce OMT , Delaine‐Smith RM , Maniati E , et al. Deconstruction of a metastatic tumor microenvironment reveals a common matrix response in human cancers. Cancer Discov. 2018;8:304‐319. 10.1158/2159-8290.CD-17-0284 PMC5837004 29196464

[142] Michelacci YM , Dietrich CP . Chondroitinase C from *Flavobacterium heparinum* . J Biol Chem. 1976;251:1154‐1158. 2603

[143] Notin P , Rollins N , Gal Y , Sander C , Marks D . Machine learning for functional protein design. Nat Biotechnol. 2024;42:216‐228. 10.1038/s41587-024-02127-0 PMC13159571 38361074

[144] Liu W , Wang Z , You R , et al. PLMSearch: protein language model powers accurate and fast sequence search for remote homology. Nat Commun. 2024;15:2775. 10.1038/s41467-024-46808-5 PMC10981738 38555371

[145] van Kempen M , Kim SS , Tumescheit C , et al. Fast and accurate protein structure search with Foldseek. Nat Biotechnol. 2024;42:243‐246. 10.1038/s41587-023-01773-0 PMC10869269 37156916

[146] Krasnow NA , Xu JA , Zhang E , et al. AI‐redesigned starting points and outcomes enhance protein evolution. Nature. 2026;657:525‐538. 10.1038/s41586-026-10820-0 PMC13477572 42486980

[147] Burke MJ , Zalewska‐Szewczyk B . Hypersensitivity reactions to Asparaginase therapy in acute lymphoblastic leukemia: immunology and clinical consequences. Future Oncol. 2022;18:1285‐1299. 10.2217/fon-2021-1288 35107320

[148] McGrath KG , Zeffren B , Alexander J , Kaplan K , Patterson R . Allergic reactions to streptokinase consistent with anaphylactic or antigen‐antibody complex‐mediated damage. J Allergy Clin Immunol. 1985;76:453‐457. 10.1016/0091-6749(85)90726-2 3928729

[149] Alewine C , Hassan R , Pastan I . Advances in anticancer immunotoxin therapy. Oncologist. 2015;20:176‐185. 10.1634/theoncologist.2014-0358 PMC4319635 25561510

[150] Sharma SK , Bagshawe KD . Antibody directed enzyme prodrug therapy (ADEPT): trials and tribulations. Adv Drug Deliv Rev. 2017;118:2‐7. 10.1016/j.addr.2017.09.009 28916498

[151] Ansari MA , Abubakar M , Ansari MM , et al. Antibody–drug conjugates in colorectal cancer: molecular design, preclinical advances, and translational challenges. Cancer Chemother Pharmacol. 2026;96:58. 10.1007/s00280-026-04906-9 PMC13219184 42207299

[152] Concolino D , Deodato F , Parini R . Enzyme replacement therapy: efficacy and limitations. Ital J Pediatr. 2018;44:120. 10.1186/s13052-018-0562-1 PMC6238252 30442189

[153] Mazor R , Pastan I . Immunogenicity of immunotoxins containing pseudomonas exotoxin a: causes, consequences, and mitigation. Front Immunol. 2020;11:1261. 10.3389/fimmu.2020.01261 PMC7333791 32695104

[154] Gabrielli F , Bernasconi E , Toscano A , et al. Side effects of immunosuppressant drugs after liver transplant. Pharmaceuticals. 2025;18:342. 10.3390/ph18030342 PMC11946649 40143120

[155] Hagerty BL , Pegna GJ , Xu J , Tai CH , Alewine C . Mesothelin‐targeted recombinant immunotoxins for solid tumors. Biomolecules. 2020;10:973. 10.3390/biom10070973 PMC7408136 32605175

[156] Liu W , Onda M , Lee B , et al. Recombinant immunotoxin engineered for low immunogenicity and antigenicity by identifying and silencing human B‐cell epitopes. Proc Natl Acad Sci U S A. 2012;109:11782‐11787. 10.1073/pnas.1209292109 PMC3406843 22753489

[157] Schmohl JU , Todhunter D , Oh S , Vallera DA . Mutagenic deimmunization of diphtheria toxin for use in biologic drug development. Toxins. 2015;7:4067‐4082. 10.3390/toxins7104067 PMC4626721 26473923

[158] Zinsli LV , Stierlin N , Loessner MJ , Schmelcher M . Deimmunization of protein therapeutics – recent advances in experimental and computational epitope prediction and deletion. Comput Struct Biotechnol J. 2021;19:315‐329. 10.1016/j.csbj.2020.12.024 PMC7779837 33425259

[159] United States Pharmacopeia . Alteplase. USP‐NF2017. 10.31003/USPNF_M1670_03_01.

[160] Pham NB , Meng WS . Protein aggregation and immunogenicity of biotherapeutics. Int J Pharm. 2020;585:119523. 10.1016/j.ijpharm.2020.119523 PMC7362938 32531452

[161] Marton C , Clémenceau B , Dachy G , et al. Harmonisation of quality control tests for academic production of CAR‐T cells: a position paper from the WP‐bioproduction of the UNITC consortium. Bone Marrow Transplant. 2025;60:1209‐1217. 10.1038/s41409-025-02637-8 PMC12401719 40442256

[162] European Medicines Agency . Guideline on Quality, Non‐clinical and Clinical Requirements for Investigational Advanced Therapy Medicinal Products in Clinical Trials. European Medicines Agency; 2025.

[163] Gómez‐Melero S , Hassouneh F , Vallejo‐Bermúdez IM , Agüera‐Morales E , Solana R , Caballero‐Villarraso J . Tandem CAR‐T cell therapy: recent advances and current challenges. Front Immunol. 2025;16:1546172. 10.3389/fimmu.2025.1546172 PMC11907001 40092990

[164] US Food and Drug Administration CfBEaR . Potency assurance for cellular and gene therapy products: Draft guidance for industry. 2023. https://www.fda.gov/regulatory‐information/search‐fda‐guidance‐documents/potency‐assurance‐cellular‐and‐gene‐therapy‐products.

